# Immunometabolic Stratification of Autism Spectrum Disorder by CD4^+^ T-Cell Phenotype Reveals Subtype-Specific Energetic Deficit and Coordinated Suppression of Micronutrient Acquisition Pathways

**DOI:** 10.3390/metabo16060416

**Published:** 2026-06-15

**Authors:** Albion Dervishi

**Affiliations:** Anaesthesiology and Intensive Care Medicine, Medius Clinic Nürtingen-Academic Teaching Hospital, University of Tübingen, Auf dem Säer 1, 72622 Nürtingen, Germany; albiondervishi@gmail.com; Tel.: +49-7022-78-22208

**Keywords:** autism spectrum disorder, immunometabolic stratification, CD4^+^ T-cell subtypes, Global Gap, τ-axis, Warburg effect, folate transport, vitamin B12, vitamin D, micronutrient triage, luxury pathway suppression, GPL570, GPL6244

## Abstract

Background: Autism spectrum disorder (ASD) is associated with immune dysregulation in a subset of individuals, though findings remain heterogeneous and poorly defined, particularly regarding immune subtypes and metabolic context. Methods: We analyzed whole-blood microarray data from GSE18123 (GPL570: ASD n = 46, controls n = 19; GPL6244: ASD n = 68, controls n = 21) using an integrated immunometabolic framework. CD4^+^ T-cell transcriptional programs were used to assign dominant immune phenotypes (TH1, TH2, TH17, Tfh, FOXP3^+^ Treg, Tr1-like). Metabolic demand was quantified via the τ-axis; execution capacity was assessed using cytosolic and mitochondrial energy compensation ratios (CECR, MECR). Induction–execution mismatch was captured by three Gap metrics (Cytosolic, Warburg, Global). Functional validation correlated these metrics with transcriptional signatures of folate transport, one-carbon metabolism, receptor-mediated micronutrient uptake (LRP2–CUBN–AMN), cobalamin processing, and vitamin D activation across both platforms. Results: Six immunometabolic CD4^+^ subtypes were identified within ASD. τ-axis discrimination was strongest for Tr1-like (AUC = 0.811) and Tfh (AUC = 0.825) states, while TH17 profiles were indistinguishable from controls. Despite variation in metabolic demand, CECR and MECR remained relatively preserved, indicating decoupling between induction and execution capacity. Global Gap values were most negative in Tfh and TH1 states and positive in TH17 and controls. Negative Gap states showed coordinated suppression of ATP-intensive micronutrient acquisition pathways, including folate transport (FOLR1/2, SLC19A1), megalin–cubilin-mediated uptake (r ≈ 0.77–0.79), and vitamin D activation (CYP27B1). Intracellular cobalamin processing was upregulated in proportion to metabolic demand (r > 0.9). Findings were directionally replicated across both datasets. Conclusions: These data demonstrate that ASD exhibits structured immunometabolic heterogeneity characterized by subtype-specific demand–capacity imbalance. The Global Gap framework provides transcriptomic evidence of energetic deficit in Tfh- and Tr1-like-dominant states. Future clinical studies should incorporate subtype-stratified assessment of micronutrient status and metabolic execution capacity.

## 1. Introduction

Autism spectrum disorder (ASD) is a heterogeneous neurodevelopmental condition increasingly associated with systemic immune and metabolic dysregulation. Multiple studies have reported abnormalities in inflammatory cytokines, immune-cell activation, oxidative metabolism, and mitochondrial function in subsets of individuals with ASD [1,2,3,4]. However, these findings remain highly variable across studies, and no unified framework currently explains how immune-state organization relates to metabolic function within ASD.

Recent work in immunometabolism has demonstrated that immune-cell activation requires coordinated metabolic adaptation involving glycolysis, mitochondrial respiration, and anabolic pathway engagement [4,5,6]. In activated immune states, metabolic demand may exceed execution capacity—a phenomenon termed energetic dysfunction—creating functional deficit despite apparent pathway induction [7,8]. Conventional transcriptomic analyses, however, typically evaluate pathway activation in isolation and do not assess the relationship between metabolic demand and execution adequacy.

At the same time, emerging evidence suggests that nutrient transport and micronutrient handling may be altered during inflammatory activation. Experimental studies have shown that inflammatory signaling and lipopolysaccharide exposure can suppress multiple vitamin transport systems, including biotin, thiamine, and ascorbate transport pathways [9,10,11]. Whether similar coordinated suppression of micronutrient acquisition occurs within ASD-associated immunometabolic states remains unknown.

Elevated TCA cycle intermediates—including succinate and 2-oxoglutaric acid—have been reported in urine of children with ASD, consistent with mitochondrial substrate-level stress (though microbial contributions cannot be excluded) [12,13], suggesting systemic metabolic stress. Together, these observations motivated an integrated immunometabolic framework combining CD4^+^ T-cell transcriptional program scoring with derived metrics of metabolic demand and execution capacity, applied to whole-blood transcriptomic data. We asked whether dominant CD4^+^ immune states in ASD reflect a shared systemic immunometabolic program, and whether high-demand states exhibit coordinated suppression of ATP-intensive micronutrient acquisition pathways—specifically folate transport, megalin–cubilin-mediated endocytosis, and vitamin D activation—as a functional consequence of energetic deficit.

## 2. Methods

### 2.1. Microarray Data Processing and Cohort Selection

The publicly available autism microarray dataset GSE18123 was retrieved from the Gene Expression Omnibus (GEO), comprising 170 individuals with autism spectrum disorder (ASD) and 115 controls [14]. Preprocessed, background-corrected expression data in MAS5-normalized format were obtained as provided by the original study.

Probe identifiers were annotated to Ensembl gene identifiers (ENSG) using the hgu133plus2.db annotation package (v3.13.0, Bioconductor). For genes represented by multiple probes, the probe with the highest mean expression across all samples was selected to ensure a single representative value per gene.

Samples from the GPL570 platform (66 male ASD and 33 age-matched male controls) and the GPL6244 platform (104 ASD and 82 controls) were processed independently due to differences in microarray design and probe composition.

Diagnostic categories included ASD, Pervasive Developmental Disorder–Not Otherwise Specified (PDD-NOS), Asperger’s Disorder, and controls. Individuals diagnosed with ASD and PDD-NOS were grouped as ASD, while those with Asperger’s Disorder were excluded (n = 23).

To reduce potential confounding, individuals with known genetic syndromes were excluded from both the ASD and control groups. Control subjects were screened to exclude attention-deficit/hyperactivity disorder (ADHD), other neurodevelopmental conditions, and systemic diseases. Demographic matching was performed to ensure consistency between ASD and control groups, particularly with respect to ethnicity, as the majority of ASD participants were of Caucasian origin.

After applying inclusion and exclusion criteria, the final sample sizes were GPL570 (ASD, n = 46; controls, n = 19) and GPL6244 (ASD, n = 68; controls, n = 21). Expression values were log_2_-transformed prior to downstream analyses.

Mapping of the 21 immunometabolic modules and CD4^+^ classifier genes to GPL570 and GPL6244 confirmed complete coverage of all 247 genes on both platforms (Supplementary Figure S1).

### 2.2. Immune–Metabolic Subtyping Within the ASD Cohort

To identify dominant immune–metabolic states within the ASD cohort, CD4^+^ T-cell transcriptional programs were scored for each sample. Curated gene signatures were used to represent six CD4^+^ phenotypes: TH1, TH2, TH17, T follicular helper cells (Tfh), FOXP3^+^ regulatory T cells (Treg), and Tr1-like regulatory cells. These signatures included lineage-defining transcription factors, cytokines, receptors, and effector molecules, such as TBX21, IFNG, STAT1, and CXCR3 for TH1; GATA3, IL4, IL5, IL13, and STAT6 for TH2; RORC, IL17A, IL17F, IL23R, and CCR6 for TH17; BCL6, CXCR5, PDCD1, ICOS, and IL21 for Tfh; FOXP3, IL2RA, CTLA4, IKZF2, and TIGIT for FOXP3^+^ Treg; and IL10, LAG3, HAVCR2, MAF, and PRDM1 for Tr1-like cells. The complete CD4^+^ subtype gene sets are provided in Supplementary Files S1 and S2.

For each subtype, a sample-level score was calculated as the mean expression of genes present in the corresponding signature. Subtype scores were then standardized across samples to obtain z-scores. Control samples were retained as a single reference group and were not assigned to CD4^+^ immune subtypes.

**Figure 1:**
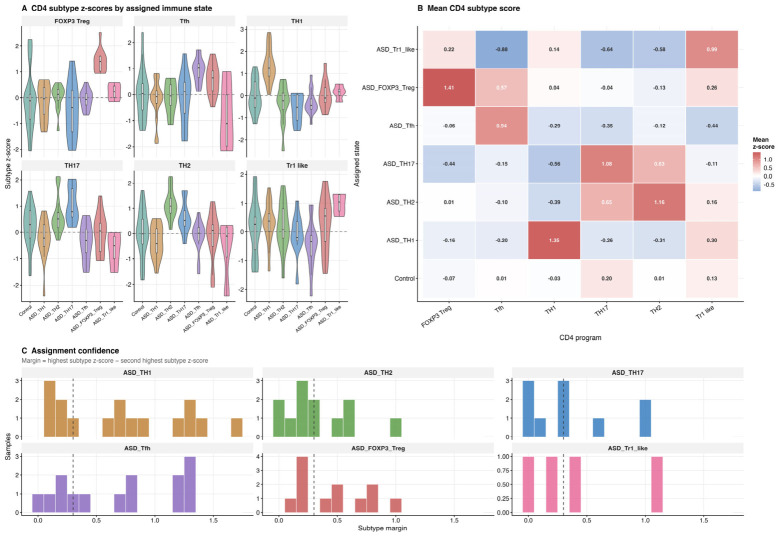
CD4^+^ phenotype selection defines distinct immune-state architecture. CD4^+^ immune-state assignment was performed using standardized transcriptional subtype scores. (**A**) Violin plots showing CD4^+^ subtype z-score distributions across assigned immune states. Each ASD immune-state group shows enrichment along its corresponding subtype axis, supporting dominant transcriptional polarization. (**B**) Heatmap of mean CD4^+^ subtype z-scores across immune states, demonstrating diagonal enrichment of the defining CD4^+^ program with limited cross-lineage overlap. (**C**) Distribution of subtype assignment margins, defined as the difference between the highest and second-highest CD4^+^ subtype z-score. Larger margins indicate stronger subtype assignment confidence.

For each ASD sample, the subtype with the highest z-score was defined as the candidate dominant immune phenotype. To avoid assigning samples without clear immune polarization, subtype assignment required the leading subtype score to be positive. In the confidence-filtered analysis, the leading subtype also had to exceed the second-highest subtype score by at least 0.30 standard deviation units. Samples not meeting these criteria were considered ambiguous and excluded from immune-state-stratified downstream analyses (Figure 1).

Formally, for ASD sample i, the dominant subtype j⁎ was defined as the subtype with the maximum standardized score z_ij_. Assignment was retained only when z_ij_⁎ > 0. ASD samples not meeting this criterion were considered unassigned and excluded from immune-state-stratified analyses. Control samples were labeled as Control and were not assigned to CD4^+^ immune subtypes. Assigned ASD samples were labeled as ASD_TH1, ASD_TH2, ASD_TH17, ASD_Tfh, ASD_FOXP3_Treg, or ASD_Tr1_like, whereas control samples were labeled as Control.

Because the analysis was performed using bulk whole-blood transcriptomic data, these immune states should be interpreted as relative enrichment of CD4^+^ transcriptional programs rather than direct quantification of purified immune-cell populations.

To preserve biological independence between immune classification and metabolic scoring, genes used for CD4^+^ subtype classification were excluded from downstream metabolic module scoring.

### 2.3. τ-Axis Computational Framework

For each sample j, the τ-axis value was defined as the sum of pathway-level module scores:

$$\tau_{j}=\sum_{M\in P}S_{j}\left(M\right)$$

where P denotes the set of predefined immunometabolic pathway modules, and $S_{j}(M)$ represents the activity score of module M in sample j. This formulation allows immune and metabolic programs to jointly define the overall immunometabolic demand state without applying external weighting coefficients.

### 2.4. Gene Module Scoring

Let $E_{ij}$ denote the normalized expression value of gene i in sample j, and let M denote a gene module containing ∣M∣ genes. The module score was calculated as the mean expression of all genes within the module:

$$S_{j}\left(M\right)=\frac{1}{\left|M\right|}\sum_{i\in M}E_{i,j}$$

### 2.5. Pathway Modules

The τ-axis integrated predefined metabolic and immunoregulatory modules capturing inflammation, energy production, stress adaptation, and immunometabolic rewiring. Included modules were glycolysis, lactate dehydrogenase A (LDHA), mitochondrial substrate-level phosphorylation (mSLP), propionyl–succinyl anaplerosis (PSA), arginine–polyamine metabolism (ArgPoly), HIF-1/hypoxia response, glycolysis induction, acute inflammatory response, TNFα/NF-κB signaling, Toll-like receptor signaling, IFN-γ signaling, type I interferon signaling, IL-6/STAT3 signaling, IL-10 signaling, IL-4/TH2 signaling, inflammasome activation, pyruvate dehydrogenase (PDH), PDH control, tricarboxylic acid cycle (TCA), oxidative phosphorylation (OXPHOS), and fatty acid oxidation (FAO). Gene composition for each module is provided in Supplementary Tables S1 and S2. LDHA was retained as a single-gene module representing lactate-producing glycolytic bias, whereas ArgPoly was modeled as a multi-gene metabolic module reflecting arginine–polyamine pathway activity.

Module activity scores (M) were defined as the mean log2 expression of genes within each module. This approach follows the single-sample scoring principle established by Barbie et al. [15] and formalized in the GSVA framework [16]. However, rather than using rank-based enrichment, module scores were computed as unweighted mean expression values, which are directly compatible with the ratio-based derived metrics used in the present study.

### 2.6. Warburg-like Index and Derived Metabolic Ratios

Derived metabolic indices were computed to quantify pathway capacity and induction–response balance. For clarity, these metrics were grouped into four functional categories: (I) metabolic capacity (CECR, MECR), (II) metabolic induction (CID, MIS), (III) composite Warburg-like index, and (IV) induction–capacity mismatch (Gap metrics).

**Glycolytic capacity (CECR).** The Cytosolic Energy Compensation Ratio was defined as the glycolysis module score plus LDHA expression, divided by the summed PDH, TCA-cycle, and OXPHOS module scores:

CECR_j_ = (Glycolysis_j_ + LDHA_j_)/(PDH_j_ + TCA_j_ + OXPHOS_j_)

The numerator captures cytosolic ATP generation capacity—glycolytic flux through glucose-to-pyruvate steps combined with LDHA-mediated lactate commitment—while the denominator reflects mitochondrial oxidative capacity. This formulation directly operationalises the cytosolic-to-oxidative ATP production ratio without gene redundancy.

**Mitochondrial capacity (MECR).** The Mitochondrial Energy Compensation Ratio was defined as the sum of the mSLP and PSA module scores divided by the same mitochondrial oxidative denominator:

MECR_j_ = (mSLP_j_ + PSA_j_)/(PDH_j_ + TCA_j_ + OXPHOS_j_)

**Warburg-like index.** A composite Warburg-like index was calculated as the mean of the z-standardized glycolysis module score and z-standardized LDHA expression:

Warburg Index_j_ = [z(Glycolysis_j_) + z(LDHA_j_)]/2

**Glycolytic induction demand (CID).** Cytosolic induction demand was defined as the z-standardized glycolysis induction module score:

CID_j_ = z(Glycolysis induction_j_)

**Metabolic induction score (MIS).** MIS was defined as the sum of z-standardized glycolysis induction and TCA/OXPHOS induction scores minus the z-standardized PDH control score. Because separate PDH activation and inhibition modules were not independently defined, MIS represents a composite approximation of net metabolic induction demand across both cytosolic and mitochondrial axes:

MIS_j_ = z(Glycolysis induction_j_) + z(TCA/OXPHOS induction_j_) − z(PDH control_j_)

**Induction–execution mismatch (Gap metrics).** Three Gap indices were defined to quantify the degree to which metabolic induction demand exceeds execution capacity. Negative Gap values indicate that induction signaling outpaces execution capacity, operationally defining energetic deficit independently of absolute pathway expression levels:

Cytosolic Gap_j_ = z(CECR_j_) − CID_j_

Warburg Gap_j_ = Warburg Index_j_ − CID_j_

Global Gap_j_ = [z(CECR_j_) + z(MECR_j_)]/2 − MIS_j_

Detailed gene composition of all immunometabolic modules, module scoring procedures, normalization steps, and mathematical definitions of all derived metrics (τ-axis, CECR, MECR, Warburg Index, CID, MIS, and Gap metrics) are provided in Supplementary Material S2.

### 2.7. τ-Normalization Across Datasets

Because raw τ-values depend on expression platform and cohort-specific characteristics, τ was standardized within each dataset using z-normalization:

$$\tau_{j}^{*}=\frac{\tau_{j}-\mu_{\tau }}{\sigma_{\tau }}$$

where $\mu_{\tau }$ and $\sigma_{\tau }$ represent the mean and standard deviation of τ within the dataset. The resulting τ* values are directly comparable between independent datasets.

### 2.8. Statistical Analyses

All statistical analyses were performed in R (version 4.3.2). Gene-level differential expression was assessed using the limma package (v3.58.1) with empirical Bayes moderation. Module score comparisons between immune-state groups were conducted using Wilcoxon rank-sum tests with Benjamini–Hochberg correction for multiple comparisons. Effect sizes were calculated using Cliff’s delta (effsize v0.8.1), and multiple testing correction was applied using the Benjamini–Hochberg false discovery rate (FDR). Discriminatory performance of the τ-axis and derived metrics was assessed using receiver operating characteristic (ROC) curves and area under the curve (AUC) estimates (pROC v1.18.5). Associations between continuous variables were assessed using Spearman’s rank correlation, and group comparisons were performed using non-parametric tests as appropriate; module score associations with Global Gap and τ_z (Figures 8 and 9) used Pearson correlation. All tests were two-sided, with significance defined as FDR-adjusted *p* < 0.05. Data visualization was performed using ggplot2 (v3.5.1). Full analysis code is publicly available at: https://github.com/albiondervishi/ASD-Immunometabolic-Stratification accessed on 9 June 2026. 

## 3. Results

### 3.1. Identification of CD4^+^-Defined Immunometabolic ASD Subtypes

To determine whether immune activation in autism spectrum disorder (ASD) is associated with distinct metabolic states, we stratified ASD samples using transcriptomic signatures of canonical CD4^+^ T-cell programs. This enabled identification of biologically interpretable immune states reflecting coordinated inflammatory and regulatory activity across two independent microarray datasets (GPL570 and GPL6244).

Module-based classification revealed six dominant CD4^+^-associated subtypes across both platforms—TH1 (n = 10 GPL570; n = 16 GPL6244), TH2 (n = 6; 12), TH17 (n = 9; 10), T follicular helper (Tfh; n = 9; 14), FOXP3^+^ regulatory T cells (Treg; n = 7; 12), and Tr1-like cells (n = 5; 4)—alongside 19 and 21 age- and sex-matched controls, respectively. In total, 46 ASD samples were classified in GPL570 and 68 in GPL6244, capturing substantial immunological heterogeneity. The overall demographic characteristics of both ASD cohorts are summarized in Supplementary Table S6.

**Figure 2:**
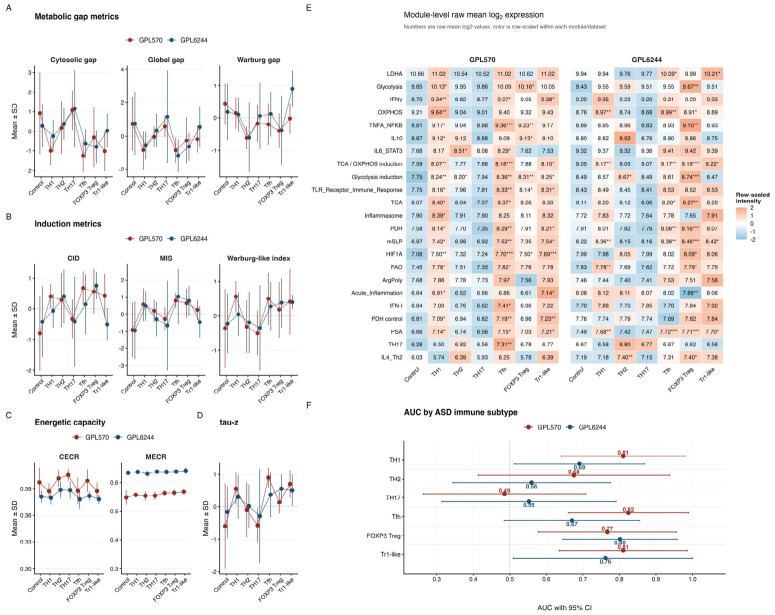
Integrated metabolic profiling of CD4^+^ T-cell-defined immune states in ASD. (**A**) Metabolic Gap metrics—Cytosolic Gap, Warburg Gap, and Global Gap—across control and ASD immune subtypes in GPL6244 and GPL570. Values are presented as mean ± SD. Negative values indicate induction–execution mismatch; positive values reflect execution capacity exceeding demand. (**B**) Induction metrics including glycolytic induction (CID), metabolic induction (MIS), and Warburg-like index. These capture activation of glycolytic and global metabolic programs and the relative bias toward aerobic glycolysis. (**C**) Energetic capacity indices—glycolytic capacity (CECR) and mitochondrial capacity (MECR)—shown on a zoomed scale to resolve subtle differences across immune states. (**D**) τ-z index representing integrated immunometabolic demand. The dashed line indicates zero, corresponding to a balanced metabolic state. (**E**) Module-level mean log_2_ expression across metabolic and immune pathways. Values are shown numerically within each cell, with color indicating row-scaled expression. Statistical significance (Wilcoxon rank-sum test, Control vs. subtype): * *p* < 0.05, ** *p* < 0.01, *** *p* < 0.001. (**F**) Classification performance (AUC with 95% CI) of the τ-axis across ASD immune subtypes. Dashed line: AUC = 0.5 (random classification).

Module-level analysis showed subtype-specific patterns of inflammatory and regulatory signaling consistent across both datasets (Figure 2E, Supplementary Tables S1–S5). Pro-inflammatory pathways, including acute inflammatory response and IFN-γ signaling, were elevated in effector-like subtypes, whereas regulatory programs were enriched in FOXP3^+^ Treg and Tr1-like states. Notably, Tr1-like samples demonstrated consistent upregulation across multiple metabolic and inflammatory modules, indicating a coordinated immunometabolic activation state replicated across platforms.

Together, these findings define a structured landscape of CD4^+^-driven immunometabolic heterogeneity in ASD, providing a reproducible framework for downstream analysis of metabolic demand, execution capacity, and induction–execution mismatch.

### 3.2. Discriminatory Performance of the τ-Axis Across CD4^+^ Subtypes

ROC analysis demonstrated subtype-specific discriminatory performance of the τ-axis across both platforms (Figure 2F, Supplementary Table S2). In GPL570, the strongest classifiers were Tfh (AUC = 0.825), TH1 (0.811), and Tr1-like (0.811), while TH17 fell below the discriminatory threshold (AUC = 0.485). In GPL6244, FOXP3^+^ Treg (AUC = 0.802) and Tr1-like (0.762) showed the most robust separation, with TH17 again performing poorly (0.552). Across both datasets, TH17 consistently showed the weakest discriminatory capacity, whereas regulatory and effector subtypes demonstrated the most reproducible τ-axis signal.

### 3.3. Coordinated and Decoupled Immunometabolic States Across ASD CD4^+^ Subtypes

To characterize immunometabolic organization across ASD immune states, we analyzed metabolic capacity, induction, and induction–capacity mismatch using complementary derived metrics (Figure 2, Supplementary Tables S3–S5).

**Metabolic capacity.** Glycolytic (CECR) and mitochondrial (MECR) capacity were broadly preserved across subtypes in both platforms (Figure 2C), with only modest variation between immune states. In contrast, the τ-axis revealed substantial differences in immunometabolic demand, highest in Tr1-like and Tfh subtypes and lowest in controls and TH17 cells (Figure 2D), indicating divergence between demand and execution capacity.

**Metabolic induction and glycolytic bias.** Induction metrics showed pronounced subtype-specific heterogeneity (Figure 2B). TH1, Tfh, and FOXP3^+^ Treg subtypes exhibited coordinated increases in Warburg-like index, CID, and MIS, consistent with aligned glycolytic and global metabolic activation. In contrast, TH2 displayed partial or less coherent induction, whereas TH17 showed uniformly low induction across all metrics, consistent with a metabolically quiescent state. Notably, Tfh cells demonstrated strong global induction despite only moderate glycolytic bias, suggesting a relative preference for mitochondrial over cytosolic activation.

**Induction–execution mismatch (Gap metrics).** Gap indices revealed pronounced subtype-specific mismatches between demand and execution (Figure 2A). Controls and TH17 showed positive Gap values across both platforms, reflecting low induction relative to preserved capacity. Tfh and TH1 exhibited strongly negative cytosolic and Global Gaps, designating these as the most metabolically constrained subtypes. FOXP3^+^ Treg showed intermediate cytosolic mismatch, while Tr1-like presented a divergent pattern between platforms, likely reflecting the small GPL6244 sample size (n = 4).

Together, these findings demonstrate that ASD immune subtypes are characterized by distinct demand–capacity relationships, with metabolic identity determined by the proportional alignment between induction and execution—a structured heterogeneity not captured by individual pathway analyses.

### 3.4. Gene-Level Metabolic Module Expression Across CD4^+^-Defined ASD Subtypes

**Figure 3:**
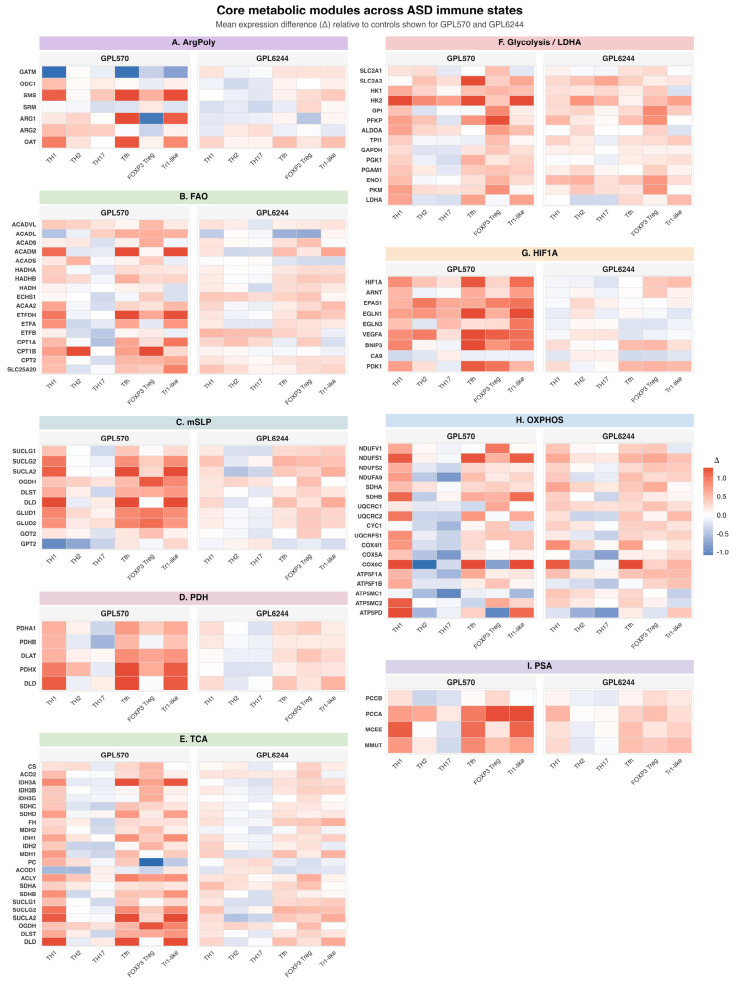
Differential expression of core metabolic modules across CD4^+^ T-cell subsets. Heatmaps show mean gene expression differences (Δ) relative to controls for selected metabolic pathways across TH1, TH2, TH17, Tfh, FOXP3^+^ Treg, and Tr1-like cells in GPL570 and GPL6244. Modules: (**A**) arginine/polyamine metabolism (ArgPoly), (**B**) fatty acid oxidation (FAO), (**C**) mitochondrial substrate-level phosphorylation (mSLP), (**D**) pyruvate dehydrogenase complex (PDH), (**E**) tricarboxylic acid (TCA) cycle, (**F**) glycolysis/LDHA, (**G**) HIF-1α signaling, (**H**) oxidative phosphorylation (OXPHOS), and (**I**) propionyl-CoA metabolism (PSA). Color scale indicates relative expression (red, upregulation; blue, downregulation). Genes are shown on the y-axis and cell subsets on the x-axis.

**Figure 4:**
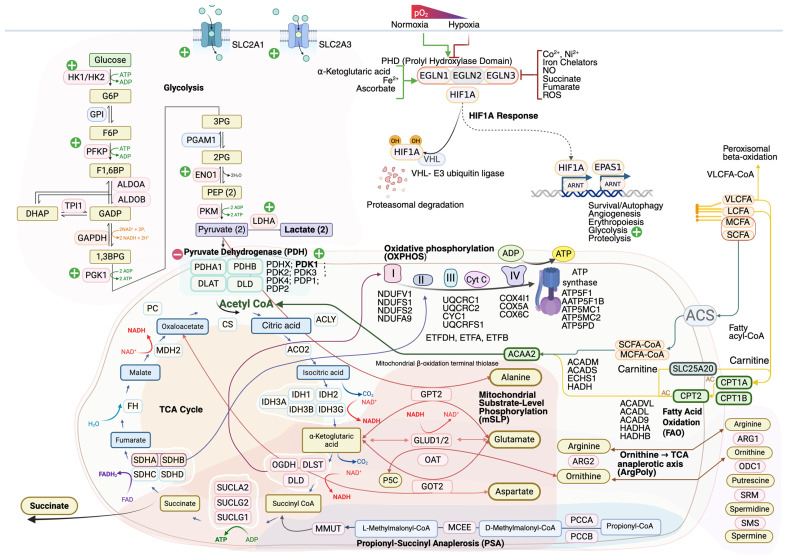
Integrated immunometabolic network underlying τ-axis regulation. Biochemical pathways visualizing the gene-level module patterns observed in Figure 3. Shown pathways include glycolysis, PDH regulation, TCA cycle, mSLP, OXPHOS, PSA, FAO, and the ornithine–TCA anaplerotic axis (Arg–polyamine pathway). The diagram integrates cytosolic and mitochondrial energy pathways and highlights the structural basis for subtype-specific metabolic programs and induction–capacity relationships. The pathway diagram was constructed manually using BioRender (Created in BioRender. Dervishi, A. (2026) https://BioRender.com/3e8qqn1, accessed on 9 June 2026), with layout informed by standard biochemical pathway conventions.

Gene-level analysis of core metabolic modules revealed coherent but subtype-specific transcriptional patterns across ASD immune states, with strong directional consistency between GPL570 and GPL6244 (Figure 3A–I), which are integrated into a unified biochemical framework in Figure 4.

Glycolysis/LDHA (Figure 3F): Effector subtypes, particularly TH1 and Tfh, showed the broadest upregulation of glycolytic genes, with HK2 and LDHA among the most consistently induced. In contrast, TH17 and control samples exhibited attenuated or near-neutral expression, consistent with low glycolytic engagement.

HIF-1α signaling (Figure 3G): HIF1A and its upstream regulators EGLN1 and EGLN3 were selectively elevated in Tfh and Tr1-like subtypes. Downstream hypoxia-responsive genes showed more variable expression, indicating partial rather than fully coordinated activation of the hypoxia program.

OXPHOS (Figure 3H): Respiratory chain genes were most elevated in Tr1-like and FOXP3^+^ Treg subtypes, with coordinated upregulation across complexes I–V. TH17 showed the most consistently reduced OXPHOS signature.

TCA cycle (Figure 3E): TCA cycle genes were most strongly upregulated in Tfh and TH1 subtypes, particularly within the succinate–succinyl-CoA axis. TH17 and TH2 showed limited or heterogeneous expression.

mSLP (Figure 3C): mSLP genes were selectively upregulated in Tfh and Tr1-like subtypes (SUCLG1, SUCLG2, OGDH), with GLUD1/2 co-induction consistent with glutamate-driven anaplerosis; TH17 showed minimal expression.

PDH (Figure 3D): PDH complex genes were modestly but consistently upregulated in TH1 and Tfh subtypes, whereas TH17 and TH2 showed near-neutral or reduced expression, suggesting limited mitochondrial pyruvate entry.

FAO (Figure 3B): FAO genes were most strongly induced in Tr1-like and FOXP3^+^ Treg subtypes, with CPT1A, ETFA, and ACADM prominently elevated. TH17 displayed uniformly reduced FAO expression.

ArgPoly (Figure 3A): Arginine/polyamine pathway genes showed selective upregulation in TH1 and Tfh subtypes, with ODC1 and SRM consistently induced. FOXP3^+^ Treg and Tr1-like subtypes showed more heterogeneous patterns.

PSA (Figure 3I): Propionyl–succinyl anaplerosis genes were modestly induced across ASD subtypes, with the most consistent elevation in Tfh and Tr1-like cells.

Together, these gene-level patterns define distinct metabolic module signatures across CD4^+^-defined ASD subtypes. Tfh and Tr1-like cells exhibited the broadest multi-pathway activation, whereas TH17 displayed the most attenuated profile. TH1 showed robust, coordinated activation, FOXP3^+^ Treg moderate but coherent induction, and TH2 an intermediate, heterogeneous pattern. These module-level patterns map directly onto the integrated biochemical network in Figure 4, providing mechanistic context for subtype-specific metabolic organization.

### 3.5. Regulatory and Induction Module Architecture Across CD4^+^-Defined ASD Subtypes

**Figure 5:**
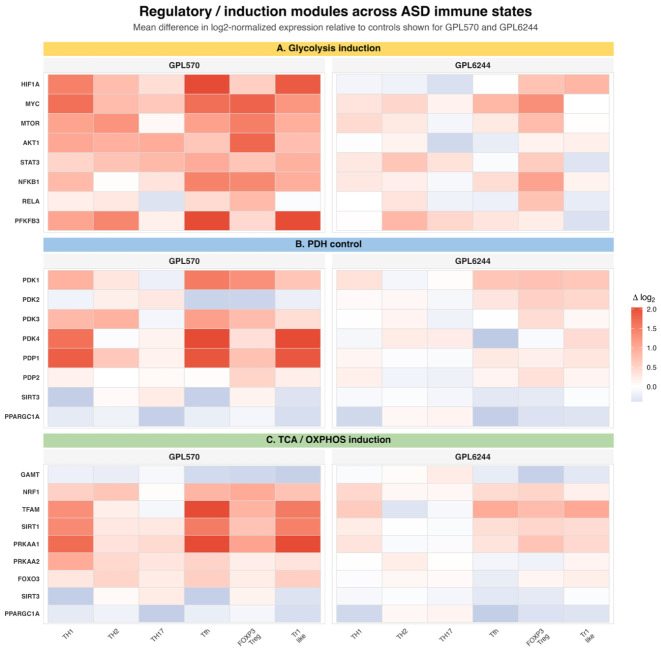
Regulatory and induction modules governing metabolic programming in CD4^+^ T-cell subsets. Heatmaps show mean gene expression differences (Δ) for key regulatory pathways across TH1, TH2, TH17, Tfh, FOXP3^+^ Treg, and Tr1-like cells in GPL570 and GPL6244. Modules include (**A**) glycolysis induction (e.g., HIF1A, MYC, MTOR), (**B**) PDH control (e.g., PDK and PDP family members), and (**C**) TCA/OXPHOS induction (e.g., NRF1, TFAM, SIRT1). Color scale represents mean expression differences (Δ) relative to controls (red, upregulation; blue, downregulation). Genes are shown on the y-axis and cell subsets on the x-axis.

To identify the upstream transcriptional mechanisms driving subtype-specific metabolic programs, we examined three regulatory modules—glycolysis induction, PDH control, and TCA/OXPHOS induction—across both platforms (Figure 5A–C).

Glycolysis induction (Figure 5A): HIF1A, MYC, and PFKFB3 were most strongly upregulated in Tfh and Tr1-like subtypes, with consistent cross-platform elevation. MTOR and AKT1 showed selective induction in Tfh and TH1, while TH17 displayed broadly attenuated upstream glycolytic regulatory signaling, consistent with its low glycolytic execution profile.

PDH control (Figure 5B): Inhibitory kinases PDK1 and PDK4 were prominently elevated in Tfh and Tr1-like subtypes, indicating active suppression of mitochondrial pyruvate entry despite high global metabolic demand. PDP1, which promotes PDH activation, was also induced in Tfh cells, suggesting a dynamic and potentially unresolved regulatory tension at the pyruvate–mitochondria interface. TH17 showed minimal PDK/PDP regulation.

TCA/OXPHOS induction (Figure 5C): TFAM, SIRT1, and PRKAA1—master regulators of mitochondrial biogenesis and energy sensing—were most strongly induced in Tfh and Tr1-like subtypes across both platforms. NRF1 and FOXO3 showed moderate elevation in FOXP3^+^ Treg, consistent with their regulatory metabolic identity. TH17 again displayed the most uniformly suppressed induction profile.

Together, these regulatory patterns provide mechanistic grounding for the subtype-specific metabolic signatures described in Section 3.4. The convergent upregulation of glycolytic inducers alongside PDH inhibitory kinases in Tfh and Tr1-like subtypes is particularly notable, suggesting a coordinated but partially blocked metabolic activation state—high induction pressure with constrained mitochondrial entry—consistent with the Gap metric findings in Section 3.3.

### 3.6. Immune Signaling Module Architecture Across CD4^+^-Defined ASD Subtypes

**Figure 6:**
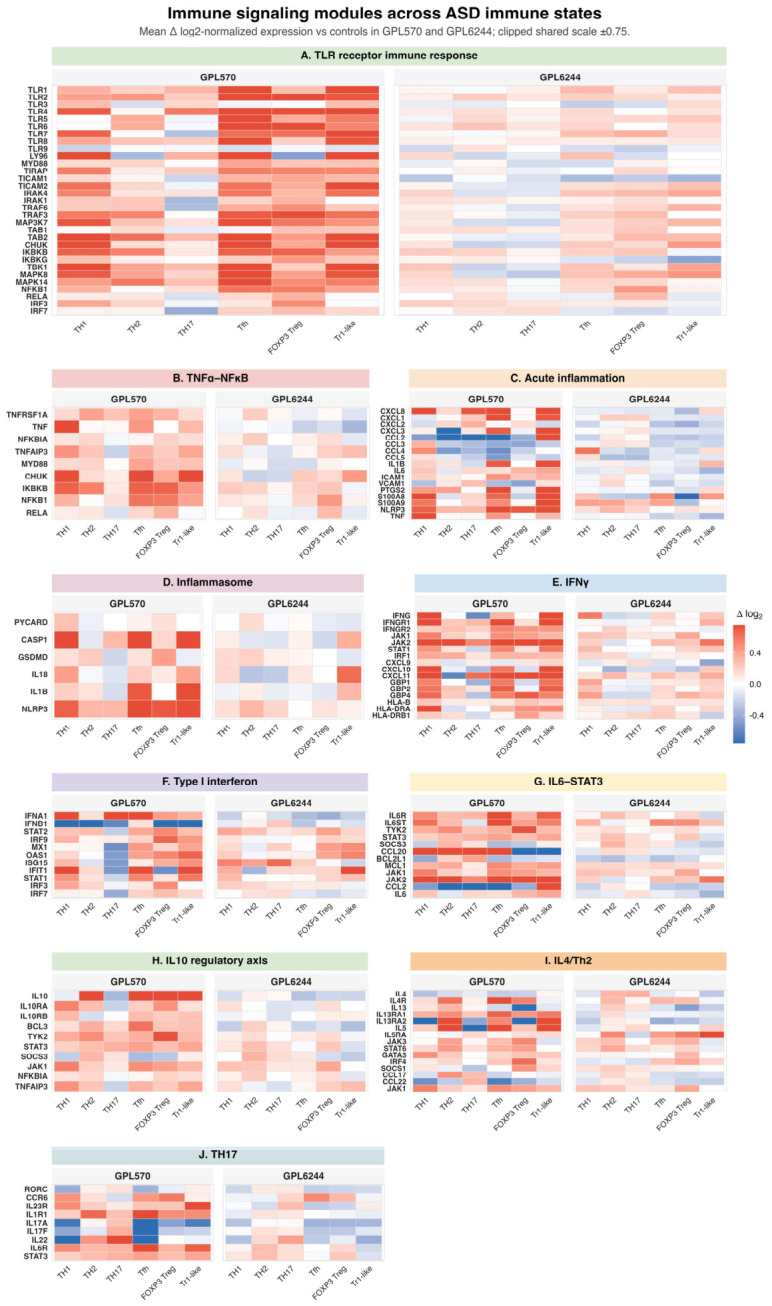
Immune signaling modules across CD4^+^-defined ASD immune states. Heatmaps display mean gene expression differences (Δlog_2_) relative to typically developing controls across six CD4^+^-defined ASD subtypes (TH1, TH2, TH17, Tfh, FOXP3^+^ Treg, Tr1-like) in GPL570 and GPL6244. Ten canonical immune signaling modules are shown: (**A**) TLR receptor immune response, (**B**) TNFα–NFκB, (**C**) acute inflammation, (**D**) inflammasome, (**E**) IFN-γ, (**F**) type I interferon, (**G**) IL-6–STAT3, (**H**) IL-10 regulatory axis, (**I**) IL-4/TH2, and (**J**) TH17. Color scale indicates mean expression change per gene relative to controls (red, upregulation; blue, downregulation; white, no change); scale is clipped at ±0.75 and shared across all modules and datasets. Gene symbols are shown on the y-axis; CD4^+^ subtypes on the x-axis.

**Figure 7:**
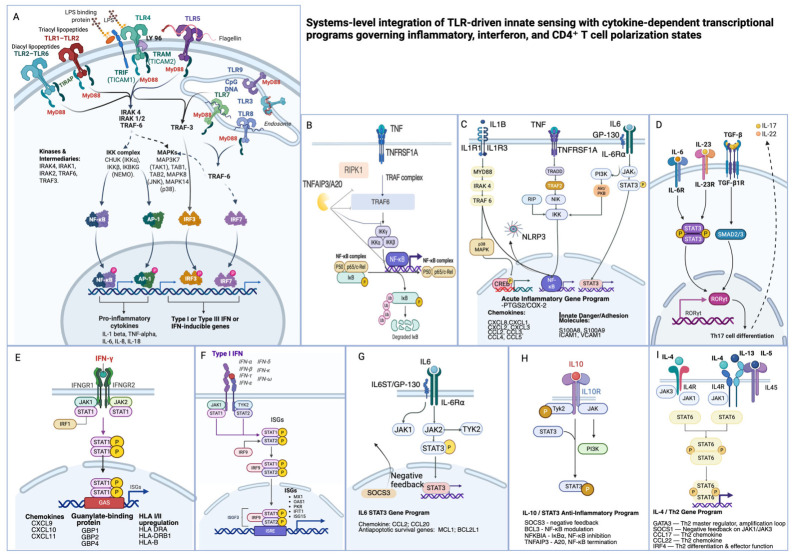
Systems-level integration of TLR-driven innate sensing with cytokine-dependent transcriptional programs governing inflammatory, interferon, and CD4^+^ T-cell polarization states. (**A**) TLR-mediated sensing of microbial ligands (LPS, lipopeptides, flagellin, nucleic acids) via surface and endosomal TLRs (TLR1/2, TLR2/6, TLR4, TLR5, TLR3, TLR7/8, TLR9) activates MyD88- and TRIF-dependent pathways, leading to IRAK/TRAF signaling, IKK/MAPK activation, and induction of NF-κB, AP-1, IRF3, and IRF7, driving pro-inflammatory cytokine and type I/III interferon responses. (**B**) TNF–TNFRSF1A signaling activates RIPK1–TRAF complexes and the IKK pathway, promoting NF-κB activation; TNFAIP3 (A20) mediates negative feedback. (**C**) Convergent IL-1β, TNF, and IL-6 signaling activates NF-κB, MAPK, and STAT3 pathways, inducing chemokines, adhesion molecules, and inflammatory mediators; NLRP3 inflammasome amplifies IL-1β signaling. (**D**) IL-6/IL-23–STAT3 signaling induces RORγt and drives TH17 differentiation (IL-17, IL-22), modulated by TGF-β/SMAD2/3. (**E**) IFN-γ activates JAK1/JAK2–STAT1 signaling, inducing GAS-dependent genes involved in antimicrobial defense, antigen presentation, and chemokine production. (**F**) Type I interferons activate JAK1/TYK2–STAT1/STAT2, forming ISGF3 (STAT1–STAT2–IRF9), which induces interferon-stimulated genes (ISGs). (**G**) IL-6–STAT3 signaling via IL-6Rα/GP-130 promotes inflammatory and survival gene expression; SOCS3 provides negative feedback. (**H**) IL-10 activates STAT3-dependent anti-inflammatory programs, including SOCS3 and TNFAIP3, and suppresses NF-κB signaling. (**I**) IL-4/IL-13 activate JAK–STAT6 signaling, inducing GATA3 and promoting Th2 differentiation and effector cytokine production. Phosphorylation events are denoted by circled P symbols. Arrows indicate activating interactions; flat-headed lines denote inhibitory relationships. Dashed lines represent indirect or multi-step signaling connections. The signaling diagram was constructed using BioRender (Created in BioRender. Dervishi, A. (2026) https://BioRender.com/mkc8rs7, accessed on 9 June 2026).

To characterize the upstream immune signaling landscape underlying CD4^+^-defined ASD states, we examined ten canonical immune modules across both platforms (Figure 6A–J). The mechanistic context for these pathways is illustrated in the integrated signaling framework in Figure 7.

TLR receptor immune response (Figure 6A and Figure 7A): TLR pathway genes were broadly upregulated across ASD subtypes, with strongest induction in TH1 and Tfh, and high cross-platform consistency.

TNFα–NFκB (Figure 6B and Figure 7B): TNF, TNFRSF1A, and RELA were selectively elevated in TH1 and Tfh, consistent with canonical NF-κB activation; co-induction of TNFAIP3 indicated concurrent negative feedback.

Acute inflammation (Figure 6C and Figure 7C): Chemokine and adhesion molecule genes (CXCL8, CCL2, ICAM1) were most strongly upregulated in TH1 and Tfh; TH17 showed heterogeneous expression.

Inflammasome (Figure 6D and Figure 7C): CASP1, IL1B, and NLRP3 were selectively elevated in TH1 and Tfh subtypes across both platforms, indicating active inflammasome engagement in effector states. FOXP3^+^ Treg and TH2 showed attenuated inflammasome signatures.

IFN-γ (Figure 6E and Figure 7E): IFNG, STAT1, and GBP family members were most strongly induced in TH1, consistent with canonical type 1 effector polarization, with strong cross-platform replication of JAK–STAT1 components.

Type I interferon (Figure 6F and Figure 7F): ISGs including MX1, OAS1, and IRF7 were selectively elevated in TH1 and Tr1-like, with consistent STAT1/STAT2 co-induction indicating ISGF3 program engagement.

IL-6–STAT3 (Figure 6G and Figure 7G): IL6R, STAT3, and SOCS3 were most elevated in Tfh and Tr1-like cells, indicating active JAK–STAT3 signaling with concurrent negative feedback.

IL-10 regulatory axis (Figure 6H and Figure 7H): IL10 and its receptor components showed selective upregulation in FOXP3^+^ Treg and Tr1-like subtypes, consistent with their regulatory identity. SOCS3 and TNFAIP3 co-induction indicates engagement of the IL-10–STAT3 anti-inflammatory program described in Figure 7H.

IL-4/TH2 (Figure 6I and Figure 7I): IL4, IL13RA1, and GATA3 were most prominently elevated in TH2 subtypes, with partial induction in FOXP3^+^ Treg. JAK–STAT6 pathway components showed consistent cross-platform upregulation in TH2, confirming canonical type 2 polarization.

TH17 (Figure 6J and Figure 7D): RORC, IL17A, and IL23R were selectively elevated in TH17 subtypes across both platforms, confirming transcriptional fidelity of the TH17 classification. Notably, IL22 and STAT3 induction was more variable, suggesting incomplete effector commitment within the TH17 group.

Together, these immune signaling patterns demonstrate that each CD4^+^-defined ASD subtype carries a distinct immune activation signature. TH1 and Tfh showed the broadest inflammatory engagement; regulatory subtypes showed selective IL-10/feedback activation; TH17 displayed a paradoxically attenuated profile despite canonical polarization markers, consistent with its metabolically quiescent state (Section 3.3, Section 3.4 and Section 3.5).

### 3.7. Global Gap and τ Associate with Coordinated Suppression of Folate Transport, Micronutrient Uptake, and Vitamin D Activation

To determine whether the Global Gap and integrated immunometabolic demand (τ_z) capture biologically meaningful cellular function beyond core metabolic pathway scoring, we evaluated their associations with three functionally linked transcriptomic systems: (i) folate transport and one-carbon metabolism, (ii) receptor-mediated micronutrient uptake, and (iii) vitamin D activation.

For each immune state, functional module scores were computed as the mean log_2_ expression difference (Δ) relative to controls across genes within each pathway. All analyses were performed independently in GPL570 and GPL6244, and concordance across platforms was used to assess robustness.

#### 3.7.1. Folate Transport and One-Carbon Metabolism

**Figure 8:**
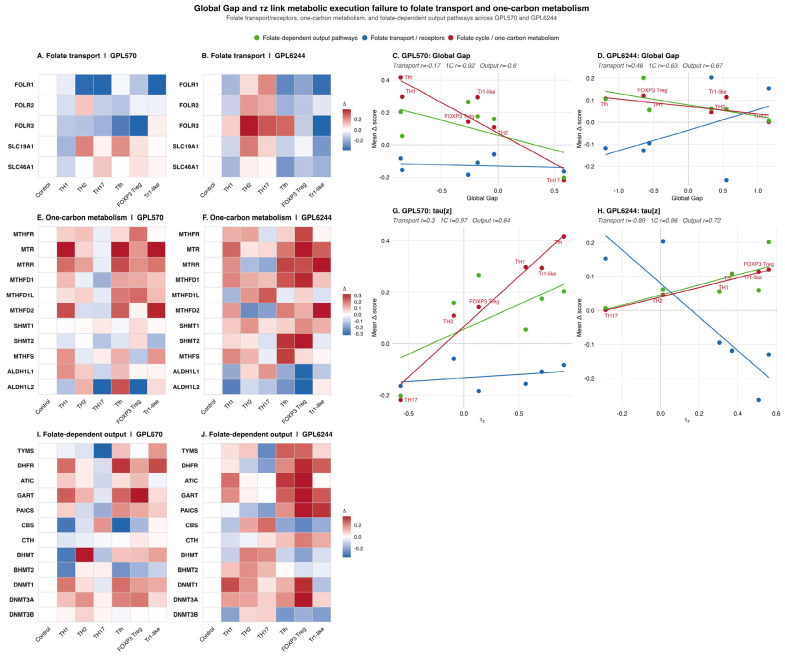
Associations between Global Gap, τ_z, and folate-related transcriptional modules across ASD CD4^+^ immune states in GPL570 and GPL6244. Heatmaps display mean gene expression differences (Δ) relative to controls (Δ = 0 baseline), contrast-scaled to the 95th percentile of absolute Δ values per panel. Red indicates upregulation; blue indicates downregulation relative to controls. Scatter plots display mean Δ module scores on the y-axis versus Global Gap (**G**,**H**) or τ z (**I**,**J**) on the x-axis, with per-module linear regression lines. Pearson correlation coefficients are shown in panel subtitles. (**A**,**B**) Folate transport and receptor genes (FOLR1, FOLR2, FOLR3, SLC19A1, SLC46A1) in GPL570 (**A**) and GPL6244 (**B**). (**C**,**D**) Folate cycle and one-carbon metabolism genes (MTHFR, MTR, MTRR, MTHFD1, MTHFD1L, MTHFD2, SHMT1, SHMT2, MTHFS, ALDH1L1, ALDH1L2) in GPL570 (**C**) and GPL6244 (**D**). (**E**,**F**) Folate-dependent output pathway genes (TYMS, DHFR, ATIC, GART, PAICS, CBS, CTH, BHMT, BHMT2, DNMT1, DNMT3A, DNMT3B) in GPL570 (**E**) and GPL6244 (**F**). (**G**,**H**) Scatter plots of Global Gap versus mean Δ scores for folate transport/receptors, folate cycle/one-carbon metabolism, and folate-dependent output pathways in GPL570 (**G**) and GPL6244 (**H**). (**I**,**J**) Scatter plots of τ_z versus mean Δ scores for the same three modules in GPL570 (**I**) and GPL6244 (**J**).

Folate receptor and transporter genes (FOLR1, FOLR2, SLC19A1) exhibited progressive suppression in immune states with increasingly negative Global Gap values, most prominently in Tfh- and Tr1-like-dominant subtypes (Figure 8A,B). In contrast, expression was relatively preserved or elevated in TH17 and TH2 states characterized by neutral or positive Gap values (Figure 8A,B). This directional pattern was consistent across both platforms.

Within the one-carbon metabolism module, a clear compartmental dissociation emerged (Figure 8C,D). Mitochondrial enzymes (MTHFD2, SHMT2) were upregulated in high-demand, negative-Gap states, whereas cytosolic folate-processing genes, including ALDH1L1, showed broad suppression (Figure 8C,D). Among folate-dependent downstream pathways, transsulfuration genes (CBS, CTH) were reduced in negative-Gap states, while nucleotide synthesis genes (TYMS, DHFR) remained relatively elevated, consistent with sustained proliferative or biosynthetic demand (Figure 8E,F).

The one-carbon metabolism module score showed a strong inverse correlation with Global Gap (GPL570: r = −0.92; GPL6244: r = −0.63; Figure 8G,H) and a strong positive correlation with τ_z (GPL570: r = 0.97; GPL6244: r = 0.96; Figure 8I,J), indicating that mitochondrial one-carbon flux scales tightly with total immunometabolic demand across platforms.

#### 3.7.2. Receptor-Mediated Micronutrient Uptake

**Figure 9:**
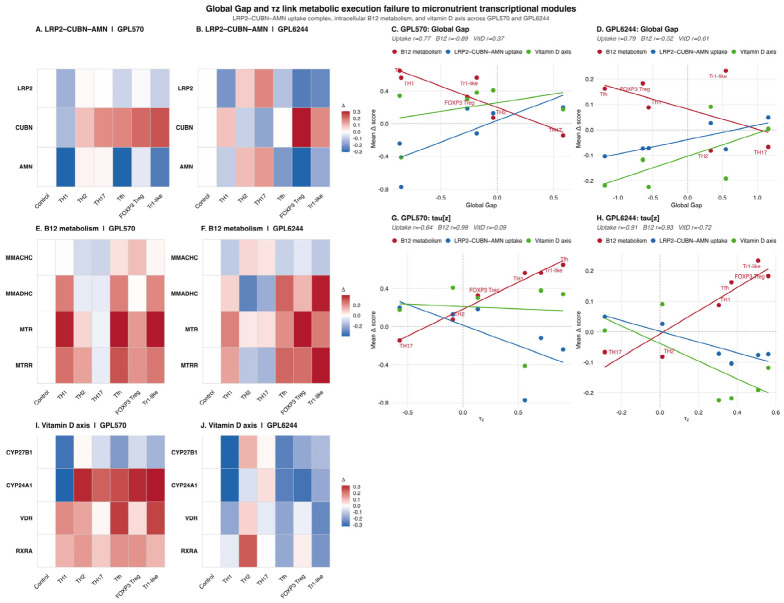
Associations between Global Gap, τ_z, and micronutrient-related transcriptional modules across ASD CD4^+^ immune states in GPL570 and GPL6244. Heatmaps display mean gene expression differences (Δ) relative to controls (Δ = 0 baseline), contrast-scaled to the 95th percentile of absolute Δ values per panel. Red indicates upregulation; blue indicates downregulation relative to controls. Scatter plots display mean Δ module scores on the y-axis versus Global Gap (**G**,**H**) or τ z (**I**,**J**) on the x-axis, with per-module linear regression lines. Pearson correlation coefficients are shown in panel subtitles. (**A**,**B**) LRP2–CUBN–AMN megalin–cubilin–amnionless endocytic uptake complex genes in GPL570 (**A**) and GPL6244 (**B**). (**C**,**D**) Intracellular B12 metabolism genes (MMACHC, MMADHC, MTR, MTRR) in GPL570 (**C**) and GPL6244 (**D**). (**E**,**F**) Vitamin D axis genes (CYP27B1, CYP24A1, VDR, RXRA) in GPL570 (**E**) and GPL6244 (**F**). (**G**,**H**) Scatter plots of Global Gap versus mean Δ module scores for the LRP2–CUBN–AMN uptake complex, intracellular B12 metabolism, and vitamin D axis in GPL570 (**G**) and GPL6244 (**H**). (**I**,**J**) Scatter plots of τ_z versus mean Δ module scores for the same three systems in GPL570 (**I**) and GPL6244 (**J**).

The megalin–cubilin–amnionless endocytic complex (LRP2, CUBN, AMN), which mediates ATP-dependent receptor-mediated uptake of vitamin B12-transcobalamin, folate-binding proteins, and vitamin D metabolites, was suppressed in immune states with the most negative Global Gap values (Figure 9A,B). This suppression was most pronounced in Tfh- and Tr1-like-dominant subtypes and was directionally consistent across both platforms (Figure 9A,B). The uptake module score correlated positively with Global Gap (GPL570: r = 0.77; GPL6244: r = 0.79; Figure 9G,H) and negatively with τ_z (GPL570: r = −0.64; GPL6244: r = −0.91; Figure 9I,J), indicating that endocytic uptake capacity is inversely related to total immunometabolic demand.

#### 3.7.3. Intracellular B12 Metabolism

In contrast to the suppression of receptor-mediated uptake, intracellular cobalamin processing genes MMACHC, MMADHC, MTR, and MTRR were upregulated in immune states with the most negative Global Gap values (Figure 9C,D). The B12 metabolism score was negatively correlated with Global Gap (GPL570: r = −0.89; GPL6244: r = −0.52; Figure 9G,H) and strongly positively correlated with τ_z (GPL570: r = 0.99; GPL6244: r = 0.93; Figure 9I,J). The near-perfect replication of the τ_z–B12 association across independent microarray platforms constitutes the strongest cross-platform signal in this study, supporting the interpretation that upregulation of intracellular cobalamin processing represents a robust compensatory response to reduced external micronutrient acquisition under high immunometabolic demand.

#### 3.7.4. Vitamin D Axis

CYP27B1, encoding the mitochondrial hydroxylase responsible for activation of vitamin D to its hormonal form 1,25-dihydroxyvitamin D, was suppressed across most ASD immune subtypes, with the most pronounced reduction in Tfh- and Tr1-like-dominant states in GPL6244 (Figure 9E,F). CYP24A1, the principal catabolic enzyme of active vitamin D, showed relative upregulation in TH1 and TH2 subtypes, suggesting a net reduction in intracellular active vitamin D availability through combined impaired activation and enhanced catabolism (Figure 9E,F). The vitamin D axis score correlated positively with Global Gap (GPL570: r = 0.37; GPL6244: r = 0.61; Figure 9G,H) and negatively with τ_z in GPL6244 (r = −0.72; Figure 9J), with a weaker association in GPL570 (r = −0.09; Figure 9I), potentially reflecting reduced statistical power in the smaller platform cohort.

#### 3.7.5. Integrated Interpretation

Micronutrient pathway suppression was consistently associated with immunometabolic state across both the Global Gap and τ_z axes, and across three functional systems and two platforms (Figure 9G–J). This convergence indicates that triage is driven by demand magnitude rather than energetic imbalance alone—with ATP-costly acquisition functions coordinately suppressed while intracellular processing is compensatorily upregulated.

## 4. Discussion

### 4.1. Structured Immunometabolic Heterogeneity in ASD

This study demonstrates that ASD is characterized by structured immunometabolic heterogeneity rather than a uniform inflammatory phenotype. Using CD4^+^ transcriptional program-based stratification, we identified six biologically distinct immune states with reproducible patterns of metabolic demand, execution capacity, and micronutrient handling across two independent platforms (GPL570 and GPL6244).

This framework extends prior observations of immune dysregulation in ASD, which have largely described heterogeneous cytokine or pathway-level alterations [17,18,19,20] extending these observations by integrating immune identity with quantitative metabolic organization.

ASD-related immunometabolic deviation is not global: TH17 and TH2 samples were statistically indistinguishable from or only marginally discriminable from controls (AUC 0.485–0.552 and 0.560–0.675), indicating that immune polarization per se is insufficient to drive ASD-associated transcriptional deviation. The lowest discriminative capacity in regulatory and TH17 compartments parallels independent evidence that the TH1/Treg ratio—rather than TH17 expansion—distinguishes children with ASD and immune dysfunction from controls [21].

Discriminative signals were concentrated in a subset of immune states. FOXP3^+^ Treg-assigned samples showed the strongest cross-platform discrimination (AUC 0.767 and 0.802), identifying regulatory T-cell dysfunction as the most reproducible feature of ASD peripheral immune organization. Tfh- and Tr1-like-assigned samples showed high primary-platform discrimination (AUC 0.825 and 0.811) but reduced replication stability, particularly for Tr1-like states, where confidence intervals were unbounded in GPL6244. TH1 showed intermediate but consistent discrimination (AUC 0.811 and 0.690).

The prominence of FOXP3^+^ Treg deviation has mechanistic implications. Reduced Treg frequency and elevated effector-to-regulatory ratios are consistently reported in ASD [21,22,23], and defective peripheral tolerance would be expected to permit Tfh expansion and compensatory IL-10-dominant responses. This positions regulatory T-cell dysfunction as a candidate organizing axis of ASD immunometabolic heterogeneity, rather than one discriminative subtype among several.

### 4.2. The τ-Axis Reveals Demand–Capacity Decoupling

A central finding of this study is that ASD immune states differ mainly in transcriptomic immunometabolic demand rather than absolute execution capacity. Although cytosolic (CECR) and mitochondrial (MECR) capacity remained relatively stable across subtypes, the transcriptomic τ-axis identified major differences in integrated demand, with Tfh- and Tr1-like states showing the highest load.

This defines a non-canonical metabolic configuration in which strong transcriptional induction occurs without proportional execution capacity, resulting in a demand–capacity mismatch. Unlike classical immunometabolic models, where activation is accompanied by coordinated glycolytic and mitochondrial upregulation [5,24], some ASD immune states appear to operate under sustained induction pressure despite limited execution capacity.

Importantly, the τ-axis represents a transcriptomic estimate of integrated immunometabolic demand rather than a direct measure of metabolic flux or ATP production. The derived Gap metrics quantify the mismatch between transcriptomic demand and inferred execution capacity.

### 4.3. The Global Gap: Operational Evidence of Energetic Deficit

The Global Gap framework addresses a key limitation of transcriptomic analysis: increased pathway expression does not necessarily indicate sufficient metabolic function. By relating inferred execution capacity to transcriptomic demand, the Global Gap provides an operational measure of energetic adequacy.

Conceptually, this resembles physiological supply–demand models, in which adequacy depends on capacity relative to demand rather than absolute capacity alone [24]. Negative Global Gap values therefore indicate immune states in which transcriptomic demand exceeds inferred execution capacity, independent of absolute pathway expression.

Consistent with this interpretation, Tfh and TH1 states showed the most negative Global Gap values, whereas controls and TH17 states remained balanced or positive. These findings suggest that specific ASD immune states operate under relative energetic constraint rather than generalized metabolic deficiency.

### 4.4. Coordinated Compensatory Metabolic Activation in Negative-Gap States

TH1-, Tfh-, and Tr1-like-dominant states showed coordinated transcriptomic upregulation of mitochondrial and anaplerotic modules, including PSA, ArgPoly, and mSLP, despite negative Global Gap values.

This pattern suggests that high-demand immune states recruit compensatory metabolic programs to maintain energetic function under constrained execution capacity. Increased expression of propionyl–succinyl anaplerosis (PSA), arginine–polyamine metabolism (ArgPoly), and mitochondrial substrate-level phosphorylation (mSLP) is therefore compatible with partial metabolic compensation during sustained immunometabolic stress.

Although transcriptomic data cannot directly measure metabolic flux, the coexistence of elevated compensatory pathway expression with negative Global Gap values supports a model of adaptive but potentially insufficient energetic compensation in specific ASD immune states.

### 4.5. Implications of Age and Sex Variation Across CD4^+^-Defined ASD Subtypes

**Figure 10:**
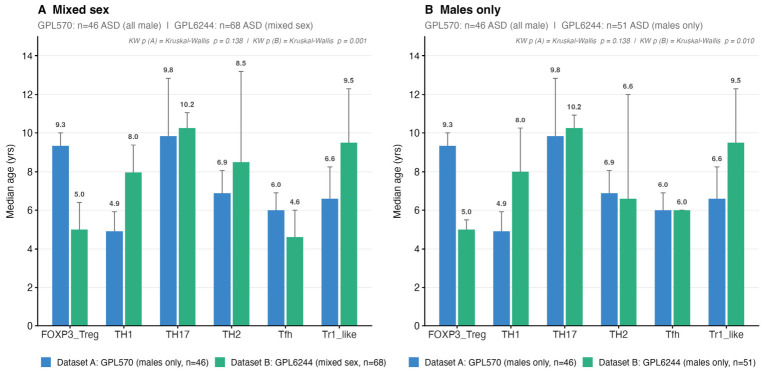
Cross-cohort comparison of age distributions across CD4^+^-defined ASD immune subtypes. Median age (years) is shown for Dataset A (GPL570; all male, n = 46 ASD) and Dataset B (GPL6244) under two conditions: (**A**) mixed-sex cohort (n = 68 ASD) and (**B**) male-only cohort (n = 51 ASD). Error bars indicate interquartile ranges (IQR). Median values are shown above each bar. Kruskal–Wallis *p*-values for age variation across subtypes are displayed within each panel.

Age distributions across CD4^+^-defined ASD subtypes were not equally reproducible between the two independent cohorts. TH17, Tfh, and TH2 subtypes showed highly comparable median ages across datasets, with agreement improving further after sex matching, whereas TH1, Tr1-like, and FOXP3^+^ Treg subtypes exhibited greater inter-cohort variability (Figure 10). This pattern suggests that certain immune states may represent relatively stable transcriptional phenotypes, while others may be more sensitive to cohort-specific biological or environmental influences. Although the present study cannot determine the underlying mechanisms, these findings are consistent with the possibility that dynamic inflammatory and regulatory immune responses contribute to subtype heterogeneity in ASD.

### 4.6. Luxury Pathway Suppression and Micronutrient Triage

The strongest transcriptional support for the Global Gap framework was the coordinated transcriptomic suppression of micronutrient acquisition and activation systems in high-demand, negative-Gap states. The most prominent suppression involved the ATP-dependent megalin–cubilin–amnionless endocytic complex (LRP2–CUBN–AMN), which mediates receptor-driven uptake of folate-, cobalamin-, and vitamin D-associated carrier complexes. Additional suppression was observed across folate transport pathways (FOLR1, FOLR2, SLC19A1) and the mitochondrial vitamin D activation enzyme CYP27B1.

Notably, vitamin D functional availability appeared transcriptionally constrained at two distinct levels: reduced intracellular substrate delivery through suppression of megalin–cubilin-mediated uptake, and reduced enzymatic activation through CYP27B1 downregulation. Together, these changes are compatible with diminished intracellular 1,25(OH)_2_D availability independent of systemic vitamin D status. These findings represent transcriptomic evidence of constrained micronutrient acquisition capacity rather than direct demonstration of functional deficiency.

In contrast, intracellular pathways involved in cobalamin processing (MMACHC, MMADHC, MTR, MTRR) and mitochondrial one-carbon metabolism (MTHFD2, SHMT2) were transcriptionally upregulated, consistent with compensatory reliance on intracellular resource utilization rather than exogenous acquisition.

These findings support a hierarchical transcriptomic metabolic response in which energetically demanding uptake systems are selectively downregulated during immunometabolic stress, while intracellular processing and core effector functions remain relatively preserved. This configuration is transcriptionally compatible with a state of metabolic triage arising under demand–capacity mismatch.

The observed suppression of micronutrient acquisition pathways is also biologically plausible in the context of inflammatory signaling, as innate immune activation has previously been shown to suppress multiple nutrient transport systems across different tissues [9,10,11,25].

This interpretation aligns conceptually with the Cell Danger Response (CDR) framework proposed by Robert Naviaux, in which chronic immune activation redirects metabolic resources away from homeostatic and anabolic processes toward defensive and damage-containment programs [26].

### 4.7. Limitations and Future Directions

This study is based on bulk transcriptomic data and therefore reflects inferred immune states rather than direct cellular or metabolic measurements. The Gap framework provides indirect systems-level evidence of metabolic imbalance, and direct validation using metabolomics, metabolic flux analysis, and single-cell approaches will be required.

Several additional limitations should also be considered. First, the dataset is cross-sectional and therefore does not allow assessment of temporal changes, developmental progression, or dynamic transitions between immune states. Second, certain ASD immune-state subgroups contained relatively small sample numbers, which may reduce statistical power and contribute to variability in subtype-specific estimates. Finally, the CD4^+^ immune-state stratification framework was derived from peripheral blood transcriptomic signatures and should therefore be interpreted as a systems-level representation of immune-transcriptional organization rather than a direct cellular immunophenotyping approach.

Future studies should integrate single-cell transcriptomics, metabolomics, microbiome profiling, and functional immune assays to further define the causal mechanisms underlying these immunometabolic states and to validate the biological relevance of the proposed Gap framework.

### 4.8. Clinical Implications: Toward Subtype-Stratified Intervention

These findings support the concept that ASD represents a heterogeneous set of immunometabolic states rather than a single biological entity. A central clinical question emerging from this framework is what drives the development of these acquisition-suppressed, high-demand immunometabolic states in specific ASD subgroups. Noninvasive metabolic assessment approaches, including urine organic acid profiling, may help identify systemic metabolic stress patterns associated with these states.

This framework suggests that acquisition-constrained ASD subtypes may respond more favorably to formulations that bypass impaired nutrient acquisition pathways than to increased substrate exposure alone. Consistent with this mechanistic reasoning, clinical studies have reported beneficial outcomes in ASD subgroups treated with folinic acid [27,28] and methylcobalamin [29,30]—forms that bypass impaired folate receptor-mediated uptake and deficient cobalamin acquisition via the megalin–cubilin endocytic complex, respectively.

Similarly, observational and interventional data suggest that vitamin D status is associated with immune-transcriptional regulation relevant to ASD neurodevelopment [31,32] and that activated vitamin D metabolites may warrant further investigation within subtype-stratified frameworks.

This is particularly relevant given that T helper cell exhaustion and immunoparalysis—states of chronic demand–capacity mismatch—remain poorly understood across many conditions. Analogous frameworks—modeling the imbalance between metabolic demand and execution capacity—may therefore prove valuable in elucidating disease mechanisms and informing subtype-stratified therapeutic approaches in a wide range of immunometabolic conditions.

## 5. Conclusions

Six CD4^+^-defined immunometabolic phenotypes were identified within ASD, each characterized by distinct patterns of metabolic demand, execution capacity, and functional micronutrient handling. The Global Gap framework demonstrated that TH1, Tfh, FOXP3^+^ Treg, and Tr1-like-dominant states exhibit a persistent induction–execution mismatch consistent with operational energetic deficit. Coordinated suppression of folate transport, megalin–cubilin uptake, and vitamin D activation pathways increased in proportion to Global Gap magnitude and was replicated across two independent transcriptomic platforms, supporting the biological relevance of this deficit. Together, these findings support a model in which ASD comprises subtype-specific immunometabolic states rather than a single uniform disorder and identify the Global Gap and τ_z—together indexing execution deficit and integrated immunometabolic demand—as transcriptomic indicators of impaired functional micronutrient acquisition in subtype-specific ASD immune states.

## Data Availability

The data presented in this study are openly available in Blood gene expression signatures distinguish autism spectrum disorders from controls at https://www.ncbi.nlm.nih.gov/geo/query/acc.cgi?acc=GSE18123 (accessed on 9 June 2026), reference number [GSE18123].

## Supplementary Materials

The following supporting information can be downloaded at: https://www.mdpi.com/article/10.3390/metabo16060416/s1. All analysis code used in this study is publicly available at: https://github.com/albiondervishi/ASD-Immunometabolic-Stratification (accessed on 9 June 2026).

## References

[1] Meltzer A., Van De Water J. (2017). The Role of the Immune System in Autism Spectrum Disorder. Neuropsychopharmacology.

[2] Li H., Xu Y., Li W., Zhang L., Zhang X., Li B., Chen Y., Wang X., Zhu C. (2023). Novel insights into the immune cell landscape and gene signatures in autism spectrum disorder by bioinformatics and clinical analysis. Front. Immunol..

[3] Gładysz D., Krzywdzińska A., Hozyasz K.K. (2018). Immune Abnormalities in Autism Spectrum Disorder—Could They Hold Promise for Causative Treatment?. Mol. Neurobiol..

[4] Morris G., Gevezova M., Sarafian V., Maes M. (2022). Redox regulation of the immune response. Cell. Mol. Immunol..

[5] Pearce E.L., Pearce E.J. (2013). Metabolic pathways in immune cell activation and quiescence. Immunity.

[6] Geltink R.I.K., Kyle R.L., Pearce E.L. (2018). Unraveling the Complex Interplay between T Cell Metabolism and Function. Annu. Rev. Immunol..

[7] Domblides C., Lartigue L., Faustin B. (2018). Metabolic stress in the immune function of T cells, macrophages and dendritic cells. Cells.

[8] Preau S., Vodovar D., Jung B., Lancel S., Zafrani L., Flatres A., Oualha M., Voiriot G., Jouan Y., Joffre J. (2021). Energetic dysfunction in sepsis: A narrative review. Ann. Intensiv. Care.

[9] Lakhan R., Said H.M. (2017). Lipopolysaccharide inhibits colonic biotin uptake via interference with membrane expression of its transporter: A role for a casein kinase 2-mediated pathway. Am. J. Physiol. Cell Physiol..

[10] Subramanian V.S., Sabui S., Moradi H., Marchant J.S., Said H.M. (2018). Inhibition of intestinal ascorbic acid uptake by lipopolysaccharide is mediated via transcriptional mechanisms. Biochim. Biophys. Acta Biomembr..

[11] Anthonymuthu S., Sabui S., Lee K., Sheikh A., Fleckenstein J.M., Said H.M. (2023). Bacterial lipopolysaccharide inhibits colonic carrier-mediated uptake of thiamin pyrophosphate: Roles for TLR4 receptor and NF-B/P38/JNK signaling pathway. Am. J. Physiol. Cell Physiol..

[12] Khan Z.U.N., Chand P., Majid H., Ahmed S., Khan A.H., Jamil A., Ejaz S., Wasim A., Khan K.A., Jafri L. (2022). Urinary metabolomics using gas chromatography-mass spectrometry: Potential biomarkers for autism spectrum disorder. BMC Neurol..

[13] Chen Q., Qiao Y., Xu X.J., Tao Y., You X. (2019). Urine organic acids as potential biomarkers for autism-spectrum disorder in chinese children. Front. Cell. Neurosci..

[14] Kong S.W., Collins C.D., Shimizu-Motohashi Y., A Holm I., Campbell M.G., Lee I.-H., Brewster S.J., Hanson E., Harris H.K., Lowe K.R. (2012). Characteristics and Predictive Value of Blood Transcriptome Signature in Males with Autism Spectrum Disorders. PLoS ONE.

[15] Barbie D.A., Tamayo P., Boehm J.S., Kim S.Y., Moody S.E., Dunn I.F., Schinzel A.C., Sandy P., Meylan E., Scholl C. (2009). Systematic RNA interference reveals that oncogenic KRAS-driven cancers require TBK1. Nature.

[16] Hänzelmann S., Castelo R., Guinney J. (2013). GSVA: Gene set variation analysis for microarray and RNA-Seq data. BMC Bioinform..

[17] Nour-Eldine W., Ltaief S.M., Abdul Manaph N.P., Al-Shammari A.R. (2022). In search of immune cellular sources of abnormal cytokines in the blood in autism spectrum disorder: A systematic review of case-control studies. Front. Immunol..

[18] Matta S.M., Hill-Yardin E.L., Crack P.J. (2019). The influence of neuroinflammation in Autism Spectrum Disorder. Brain Behav. Immun..

[19] Ashwood P., Krakowiak P., Hertz-Picciotto I., Hansen R., Pessah I., Van de Water J. (2011). Elevated plasma cytokines in autism spectrum disorders provide evidence of immune dysfunction and are associated with impaired behavioral outcome. Brain Behav. Immun..

[20] Masi A., Quintana D.S., Glozier N., Lloyd A.R., Hickie I.B., Guastella A.J. (2015). Cytokine aberrations in autism spectrum disorder: A systematic review and meta-analysis. Mol. Psychiatry.

[21] Nie Z.-Q., Han D., Zhang K., Li M., Kwon H.-K., Im S.-H., Xu L., Yang J.-C., Li Z.-W., Huang X.-W. (2023). TH1/Treg ratio may be a marker of autism in children with immune dysfunction. Res. Autism Spectr. Disord..

[22] Moreno R.J., Rose D., Ashwood P. (2026). Altered phenotype and gene expression of regulatory T cells (Tregs) in children with Autism, and the relationship with comorbid gastrointestinal symptoms. J. Neuroinflamm..

[23] Moaaz M., Youssry S., Elfatatry A., El Rahman M.A. (2019). Th17/Treg cells imbalance and their related cytokines (IL-17, IL-10 and TGF-β) in children with autism spectrum disorder. J. Neuroimmunol..

[24] Heiden M.G.V., Cantley L.C., Thompson C.B. (2009). Understanding the warburg effect: The metabolic requirements of cell proliferation. Science.

[25] Dervishi A. (2025). A Systems Hypothesis of Lipopolysaccharide-Induced Vitamin Transport Suppression and Metabolic Reprogramming in Autism Spectrum Disorders: An Open Call for Validation and Therapeutic Translation. Metabolites.

[26] Naviaux R.K. (2014). Metabolic features of the cell danger response. Mitochondrion.

[27] Frye R.E., Slattery J., Delhey L., Furgerson B., Strickland T., Tippett M., Sailey A., Wynne R., Rose S., Melnyk S. (2018). Folinic acid improves verbal communication in children with autism and language impairment: A randomized double-blind placebo-controlled trial. Mol. Psychiatry.

[28] Zhang C., Chen Y., Hou F., Li Y., Wang W., Guo L., Zhang C., Li L., Lu C. (2025). Safety and Efficacy of High-Dose Folinic Acid in Children with Autism: The Impact of Folate Metabolism Gene Polymorphisms. Nutrients.

[29] Hendren R.L., James S.J., Widjaja F., Lawton B., Rosenblatt A., Bent S. (2016). Randomized, placebo-controlled trial of methyl B12 for children with autism. J. Child Adolesc. Psychopharmacol..

[30] Čorejová A., Fazekaš T., Jánošíková D., Repiský J., Pospíšilová V., Miková M., Rauová D., Ostatníková D., Kyselovič J., Hrabovská A. (2022). Improvement of the Clinical and Psychological Profile of Patients with Autism after Methylcobalamin Syrup Administration. Nutrients.

[31] Javadfar Z., Soltani S., Khamoushi F., Sharifi M., Moradi S., Rezaeian S., Foroughi A.A., Cheshmeh S., Taghaddosi M., Bahrehmand F. (2025). Effect of vitamin D supplementation on inflammatory status and behavioral symptoms in children with autism spectrum disorders: A double-blind randomized clinical trial. BMC Pediatr..

[32] Kočovská E., Fernell E., Billstedt E., Minnis H., Gillberg C. (2012). Vitamin D and autism: Clinical review. Res. Dev. Disabil..

