# Supplementary material for: Immunometabolic Stratification of Autism Spectrum Disorder by CD4+ T-Cell Phenotype Reveals Subtype-Specific Energetic Deficit and Coordinated Suppression of Micronutrient Acquisition Pathways
Source: Metabolites. 2026 Jun 15;16(6):416. doi: 10.3390/metabo16060416 (PMC13304352; doi:10.3390/metabo16060416)
Supplement: Supplementary file 1 [file metabolites-16-00416-s001.zip › Manuscript_Supplementary Table_S1-6_ Figure.pdf]

Immunometabolic Stratification of Autism Spectrum Disorder by CD4$^+$ T Cell Phenotype Reveals Subtype-Specific Energetic Deficit and Coordinated Suppression of Micronutrient Acquisition Pathways

Albion Dervishi

Supplement Tables and Figure S1

Dataset	Module	Mean log2 Expr (Ctrl)	Mean log2 Expr (TH1)	Raw p-value (TH1)	Mean log2 Expr (TH2)	Raw p-value (TH2)	Mean log2 Expr (TH17)	Raw p-value (TH17)	Mean log2 Expr (Tfh)	Raw p-value (Tfh)	Mean log2 Expr (FOXP3_Treg)	Raw p-value (FOXP3_Treg)	Mean log2 Expr (Tr1 like)	Raw p-value (Tr1 like)
GPL6244	FAO	7,63	7,783	**0.001**	7,691	0.242	7,621	0.724	7,724	0.056	7,785	**0.016**	7,75	**0.096**
	OXPHOS	8,761	8,97	**0.003**	8,737	0.868	8,676	0.917	8,989	**0.004**	8,91	**0.040**	8,888	0.295
	PSA	7,49	7,68	**0.002**	7,419	0.274	7,47	0.492	7,716	**<0.001**	7,713	**<0.001**	7,701	**0.015**
	TCA / OXPHOS induction	9,05	9,174	**0.002**	9,053	0.897	9,066	0.217	9,172	**0.001**	9,192	**<0.001**	9,216	**0.047**
	mSLP	8,22	8,355	**0.007**	8,15	0.365	8,156	0.787	8,377	**0.003**	8,461	**<0.001**	8,418	**0.015**
	IFNγ	9,204	9,352	0.055	9,226	0.985	9,202	0.724	9,312	0.235	9,293	0.326	9,331	0.452
	Tau_index	-0,159	0,305	0.051	0,012	0.593	-0,286	0.663	0,37	0.096	0,561	**0.004**	0,508	0.113
	Glycolysis	9,434	9,554	0.089	9,589	0.089	9,51	0.327	9,546	0.145	9,674	**0.001**	9,511	0.803
	PDH	7,907	8,007	0.083	7,817	0.274	7,761	0.492	8,076	**0.006**	8,16	**<0.001**	8,073	0.096
	TCA	8,106	8,198	0.108	8,115	0.645	8,061	0.884	8,196	0.048	8,272	**0.004**	8,203	0.231
	Glycolysis induction	8,492	8,568	0.165	8,668	**0.033**	8,493	0.492	8,613	0.135	8,744	**<0.001**	8,473	1.000
	TLR Receptor Immune Response	8,428	8,495	0.195	8,452	0.868	8,411	0.724	8,53	0.210	8,521	0.152	8,535	0.452
	Inflammasome	7,723	7,827	0.254	7,716	0.542	7,638	0.546	7,781	0.829	7,652	0.200	7,91	0.262
	TH17	6,671	6,582	0.387	6,798	0.471	6,775	0.787	6,667	0.960	6,62	0.985	6,583	0.592
	IFN-I	7,699	7,88	0.457	7,728	0.956	7,854	0.519	7,7	0.987	7,842	0.593	7,92	0.748
	TNFA_NFKB	8,89	8,946	0.457	8,964	0.345	8,826	0.950	8,935	0.538	9,096	**0.005**	8,927	0.858
	IL10	8,799	8,819	0.514	8,922	0.082	8,765	0.787	8,798	0.881	8,863	0.163	8,749	0.695
	IL6_STAT3	9,324	9,366	0.575	9,315	0.671	9,363	0.852	9,407	0.309	9,416	0.345	9,39	0.748
	LDHA	9,939	9,944	0.554	9,762	0.187	9,771	0.306	10,089	**0.031**	9,986	0.427	10,206	**0.047**
	Acute_Inflammation	8,076	8,118	0.660	8,114	0.542	8,07	0.787	8,018	0.222	7,877	**0.001**	8,056	0.971
	PDH control	7,762	7,736	0.728	7,787	0.449	7,739	0.852	7,688	0.175	7,824	0.213	7,843	0.231
	ArgPoly	7,463	7,445	0.844	7,403	0.365	7,409	0.466	7,531	0.235	7,51	0.427	7,578	0.331
	HIF1A	7,992	7,975	1.000	8,054	0.187	7,994	0.950	8,019	0.414	8,089	**0.040**	8,063	0.331
	IL4_Th2	7,188	7,182	0.964	7,397	**0.009**	7,15	0.755	7,314	0.118	7,398	**0.016**	7,381	0.231
GPL570	Glycolysis induction	7,746	8,242	**0.004**	8,195	**0.030**	7,938	0.332	8,358	**0.001**	8,309	**0.006**	8,253	**0.024**
	HIF1A	7,081	7,498	**0.006**	7,316	0.400	7,243	0.410	7,697	**<0.001**	7,498	**0.022**	7,687	**<0.001**
	IFNγ	8,703	9,335	**0.003**	8,819	0.828	8,773	0.885	9,27	**0.014**	9,048	0.279	9,381	**0.024**
	OXPHOS	9,21	9,636	**0.005**	9,044	0.246	9,013	0.285	9,397	0.285	9,322	0.572	9,432	0.297
	TCA / OXPHOS induction	7,593	8,072	**0.003**	7,769	0.555	7,679	0.923	8,175	**<0.001**	7,876	0.279	8,1	**0.019**
	Tau_index	-0,596	0,56	**0.006**	-0,091	0.221	-0,573	0.923	0,906	**0.005**	0,135	**0.041**	0,706	**0.036**
	TLR Receptor Immune Response	7,748	8,165	**0.011**	7,955	0.303	7,807	0.699	8,332	0.001	8,136	**0.041**	8,307	**0.015**
	Glycolysis	9,853	10,131	**0.021**	9,95	0.687	9,863	0.885	10,094	0.068	10,163	**0.035**	10,047	0.331
	Inflammasome	7,904	8,389	**0.019**	7,907	0.687	7,9	0.629	8,252	0.076	8,112	0.188	8,316	0.160
	PDH	7,575	8,14	**0.014**	7,696	0.926	7,351	0.223	8,291	**0.004**	7,911	0.306	8,211	**0.024**
	PSA	6,661	7,143	**0.021**	6,737	0.877	6,555	0.117	7,154	**0.033**	7,033	0.135	7,205	**0.024**
	TCA	8,067	8,399	**0.019**	8,042	0.828	7,966	0.438	8,371	**0.025**	8,261	0.169	8,304	0.297
	TNFA_NFKB	8,807	9,166	**0.012**	9,042	0.156	8,88	0.595	9,361	**<0.001**	9,229	**0.007**	9,17	0.053
	mSLP	6,973	7,424	**0.019**	6,981	1.000	6,92	0.530	7,524	**0.004**	7,351	0.083	7,538	**0.036**
	FAO	7,453	7,781	**0.024**	7,507	0.926	7,35	0.383	7,818	**0.014**	7,762	0.107	7,783	0.063
	IL10	8,874	9,117	**0.031**	9,119	0.059	8,882	0.809	9,088	0.085	9,133	**0.030**	9,098	0.120
	PDH control	6,813	7,088	**0.035**	6,941	0.366	6,823	0.847	7,186	**0.005**	6,965	0.497	7,226	**0.001**
	Acute_Inflammation	6,639	6,908	**0.050**	6,523	0.926	6,661	0.629	6,86	0.172	6,607	0.910	7,14	**0.036**
	IL6_STAT3	7,683	8,174	0.104	8,514	**0.025**	8,082	0.285	8,286	**0.022**	7,615	0.821	7,53	0.836
	LDHA	10,655	11,016	0.126	10,537	0.437	10,515	0.308	11,021	0.117	10,623	0.611	11,024	0.160
	ArgPoly	7,682	7,88	0.228	7,778	0.975	7,735	0.735	7,974	0.117	7,559	0.334	7,934	0.297
	IFN-I	6,843	7,094	0.308	6,757	1.000	6,619	0.699	7,406	**0.042**	6,959	0.866	7,215	0.783
	IL4_Th2	6,033	5,737	0.456	6,39	0.475	5,93	0.438	6,253	0.847	5,784	0.955	6,393	0.629
	TH17	6,283	6,505	0.604	6,922	0.106	6,56	0.410	7,309	0.002	6,778	0.152	6,772	0.208

Supplementary Table S1 | Module-level gene expression across CD4$^+$ T cell–defined ASD immune states.

Module scores represent mean $\log_2$-transformed expression values ($\log_2$-CPM) for control samples and ASD immune subtypes (TH1, TH2, TH17, Tfh, FOXP3$^+$ Treg, and Tr1-like cells) across the GPL6244 and GPL570 datasets. For each module, control values are shown alongside subtype-specific means.

Statistical comparisons were performed using two-sided Wilcoxon rank-sum tests (Control vs. each ASD subtype). Raw p-values are reported for each comparison.
Modules correspond to curated metabolic and immune pathways, including oxidative phosphorylation (OXPHOS), fatty acid oxidation (FAO), glycolysis, TCA cycle activity, and inflammatory signaling pathways.
These module-level expression values constitute the primary data used to derive higher-order energetic indices, including CECR, MECR, Warburg-like index, glycolytic induction (CID), and related metabolic metrics presented in the main figures.
Note: Values are shown as mean $\log_2$ expression without scaling. Full datasets are provided to enable independent recalculation of all derived metrics.

	Comparison	AUC	CI low	CI high
GPL576	Control vs. ASD_TH1	0,81052632	0,6413379	0,97971473
	Control vs. ASD_TH2	0,6754386	0,41358024	0,93729695
	Control vs. ASD_TH17	0,48538012	0,26275068	0,70800956
	Control vs. ASD_Tfh	0,8245614	0,66010941	0,98901339
	Control vs. ASD_FOXP3_Treg	0,76691729	0,57762261	0,95621198
	Control vs. ASD_Tr1_like	0,81052632	0,63479069	0,98626195
GPL6249	Control vs. ASD_TH1	0,69047619	0,511614	0,869339
	Control vs. ASD_TH2	0,55952381	0,344022	0,775026
	Control vs. ASD_TH17	0,55238095	0,31396	0,790802
	Control vs. ASD_Tfh	0,67006803	0,484619	0,855517
	Control vs. ASD_FOXP3_Treg	0,8015873	0,64538	0,957794
	Control vs.ASD_Tr1_like	0,76190476	0,509665	1

Supplementary Table S2| Discriminatory performance of the τ-axis across CD4$^+$-defined ASD subtypes.

Area under the receiver operating characteristic curve (AUC) and 95% confidence intervals (CI) for pairwise comparisons between typically developing controls and each CD4$^+$-defined ASD immune subtype, computed independently for the GPL570 and GPL6244 microarray datasets. Higher AUC values indicate stronger separation of ASD subtype from controls along the τ-axis. CI limits were estimated by bootstrapping. Subtypes: TH1, T helper 1; TH2, T helper 2; TH17, T helper 17; Tfh, T follicular helper; FOXP3$^+$ Treg, FOXP3-expressing regulatory T cells; Tr1-like, type 1 regulatory T-like cells.

Dataset	Immune State	n	Glycolytic capacity (CECR)	Mitochondrial capacity (MECR)	τ-z
GPL6244	Control	21	0.381 ± 0.007	0.634 ± 0.006	-0.159 ± 0.789
	ASD TH1	16	0.380 ± 0.007	0.637 ± 0.006	0.305 ± 0.660
	ASD TH2	12	0.389 ± 0.009	0.631 ± 0.008	0.012 ± 0.665
	ASD TH17	10	0.388 ± 0.010	0.638 ± 0.005	-0.286 ± 1.444
	ASD Tfh	14	0.378 ± 0.010	0.637 ± 0.008	0.370 ± 0.676
	ASD FOXP3 Treg	12	0.382 ± 0.005	0.638 ± 0.006	0.561 ± 0.427
	ASD Tr1 like	4	0.378 ± 0.005	0.641 ± 0.010	0.508 ± 0.477
GPL570	Control	19	0.397 ± 0.016	0.548 ± 0.024	-0.596 ± 1.311
	ASD TH1	10	0.387 ± 0.009	0.557 ± 0.008	0.560 ± 0.483
	ASD TH2	6	0.402 ± 0.009	0.553 ± 0.015	-0.091 ± 0.836
	ASD TH17	9	0.406 ± 0.007	0.554 ± 0.013	-0.573 ± 0.520
	ASD Tfh	9	0.388 ± 0.014	0.563 ± 0.008	0.906 ± 0.293
	ASD FOXP3 Treg	7	0.399 ± 0.012	0.564 ± 0.011	0.135 ± 0.327
	ASD Tr1 like	5	0.387 ± 0.009	0.568 ± 0.008	0.706 ± 0.411

Supplementary Table S3 | Energetic capacity indices across CD4⁺ T cell–defined ASD immune states.

Glycolytic capacity (CECR), mitochondrial capacity (MECR), and the composite τ-z index are reported for control samples and ASD immune subtypes (TH1, TH2, TH17, Tfh, FOXP3⁺ Treg, and Tr1-like cells) across the GPL6244 and GPL570 datasets. Values are presented as mean ± standard deviation (SD), with corresponding sample sizes (n) for each group. CECR and MECR quantify relative glycolytic and mitochondrial energetic capacity, respectively, derived from module-level gene expression profiles. The τ-z index represents the integrated balance between glycolytic and mitochondrial programs, expressed as a standardized composite metric. These indices are directly derived from the module-level expression data provided in Supplementary Table 1 using the predefined computational framework described in the Methods.

Note: Values are reported in absolute units without normalization to control, enabling direct comparison across immune states within each dataset.

Dataset	Immune State	n	Warburg-like index	Glycolytic induction (CID)	Metabolic induction (MIS)
GPL6244	Control	21	-0.231 ± 0.857	-0.418 ± 1.139	-0.941 ± 1.661
	ASD TH1	16	0.043 ± 0.613	-0.064 ± 0.545	0.483 ± 0.836
	ASD TH2	12	-0.189 ± 0.875	0.404 ± 0.865	-0.283 ± 0.966
	ASD TH17	10	-0.349 ± 1.222	-0.414 ± 1.449	-0.655 ± 2.615
	ASD Tfh	14	0.271 ± 0.690	0.146 ± 0.958	1.028 ± 1.598
	ASD FOXP3_Treg	12	0.380 ± 0.701	0.758 ± 0.439	0.785 ± 0.847
	ASD Tr1_like	4	0.394 ± 0.193	-0.508 ± 0.506	-0.457 ± 0.889
GPL570	Control	19	-0.360 ± 1.127	-0.792 ± 1.203	-0.912 ± 1.576
	ASD TH1	10	0.554 ± 0.447	0.403 ± 0.388	0.572 ± 0.934
	ASD TH2	6	-0.315 ± 0.747	0.291 ± 0.874	0.194 ± 0.611
	ASD TH17	9	-0.499 ± 0.636	-0.329 ± 0.615	-0.276 ± 0.644
	ASD Tfh	9	0.490 ± 0.745	0.683 ± 0.530	0.796 ± 0.418
	ASD FOXP3_Treg	7	0.174 ± 0.799	0.565 ± 0.721	0.647 ± 0.832
	ASD Tr1_like	5	0.409 ± 0.934	0.430 ± 0.709	0.247 ± 0.571

Supplementary Table S4 | Metabolic induction and Warburg-related indices across CD4⁺ T cell–defined ASD immune states.

Warburg-like index, glycolytic induction (CID), and metabolic induction (MIS) are reported for control samples and ASD immune subtypes (TH1, TH2, TH17, Tfh, FOXP3⁺ Treg, and Tr1-like cells) across the GPL6244 and GPL570 datasets. Values are presented as mean ± standard deviation (SD), with corresponding sample sizes (n) for each group. The Warburg-like index reflects the relative shift toward aerobic glycolysis versus mitochondrial metabolism. Glycolytic induction (CID) quantifies activation of glycolytic programs, whereas metabolic induction (MIS) represents the integrated upregulation of metabolic pathways. These indices are derived from module-level $\log_2$ expression values provided in Supplementary Table 1 using the predefined computational framework described in the Methods. Together with CECR, MECR, and τ-z (Supplementary Table 2), these metrics define the metabolic state and activation profile of each immune subtype.

Note: Values are reported in absolute units without normalization to control, enabling direct comparison of metabolic induction across immune states within each dataset.

Dataset	Immune State	n	Cytosolic gap	Warburg gap	Global gap
GPL6244	Control	21	0.277 ± 1.076	0.187 ± 0.862	0.741 ± 1.866
	ASD TH1	16	-0.230 ± 0.790	0.107 ± 0.504	-0.560 ± 0.808
	ASD TH2	12	0.378 ± 0.781	-0.593 ± 1.052	0.332 ± 0.992
	ASD TH17	10	1.152 ± 1.928	0.066 ± 1.011	1.157 ± 2.753
	ASD Tfh	14	-0.626 ± 0.732	0.125 ± 0.675	-1.189 ± 0.946
	ASD FOXP3 Treg	12	-0.797 ± 0.833	-0.379 ± 1.049	-0.643 ± 0.812
	ASD Tr1 like	4	0.034 ± 0.841	0.903 ± 0.545	0.548 ± 1.204
GPL 570	Control	19	0.925 ± 2.071	0.433 ± 0.628	0.738 ± 1.597
	ASD TH1	10	-0.997 ± 0.709	0.152 ± 0.466	-0.850 ± 1.133
	ASD TH2	6	0.181 ± 1.313	-0.606 ± 0.342	-0.036 ± 0.578

ASD TH17	9	1.076 ± 0.978	-0.170 ± 0.556	0.583 ± 0.591
ASD Tfh	9	-1.249 ± 0.800	-0.192 ± 0.619	-0.864 ± 0.770
ASD FOXP3 Treg	7	-0.295 ± 1.507	-0.391 ± 0.521	-0.265 ± 1.352
ASD Tr1 like	5	-1.018 ± 0.967	-0.021 ± 0.451	-0.182 ± 0.793

Supplementary Table S5 | Metabolic gap indices across CD4^{+} T cell–defined ASD immune states.

Cytosolic gap, Warburg gap, and global metabolic gap are reported for control samples and ASD immune subtypes (TH1, TH2, TH17, Tfh, FOXP3^{+} Treg, and Tr1-like cells) across the GPL6244 and GPL570 datasets. Values are presented as mean ± standard deviation (SD), with corresponding sample sizes (n) for each group. The cytosolic gap reflects the imbalance within cytosolic metabolic programs, primarily capturing divergence in glycolytic and related pathways. The Warburg gap quantifies the relative difference between glycolytic and mitochondrial metabolic activity, serving as an indicator of aerobic glycolytic bias. The global gap represents the integrated metabolic disequilibrium across cytosolic and mitochondrial compartments. These gap indices are derived from module-level $\log_2$ expression values (Supplementary Table 1) using the predefined computational framework described in the Methods. In combination with energetic capacity (CECR, MECR, τ-z; Supplementary Table 2) and metabolic induction metrics (Warburg-like index, CID, MIS; Supplementary Table 3), these measures provide a comprehensive description of metabolic state and imbalance across immune subtypes.

Note: Values are reported in absolute units without normalization to control, enabling direct comparison of metabolic imbalance across immune states within each dataset.

	Dataset A — GPL570			Dataset B — GPL6244		
	ASD	Control	Total	ASD	Control	Total
N	46	19	65	68	21	89
Age, median [IQR], years	6.6 [5.0–8.3]	4.9 [3.6–12.8]	6.3 [4.2–9.1]	6.0 [5.0–9.3]	7.0 [6.0–11.0]	7.0 [5.0–10.0]
Male, n (%)	47 (100.0%)	19 (100.0%)	66 (100.0%)	51 (75.0%)	15 (71.4%)	66 (74.2%)
Female, n (%)	0 (0.0%)	0 (0.0%)	0 (0.0%)	17 (25.0%)	6 (28.6%)	23 (25.8%)

Dataset A (GPL570) is exclusively male by study design. Dataset B (GPL6244) includes both sexes; a male-only subanalysis (n=51 ASD, n=15 Control) was performed for cross-cohort comparability (see Supplementary Figure S1).

Supplementary Table S6. General demographic characteristics of the two independent ASD cohorts.

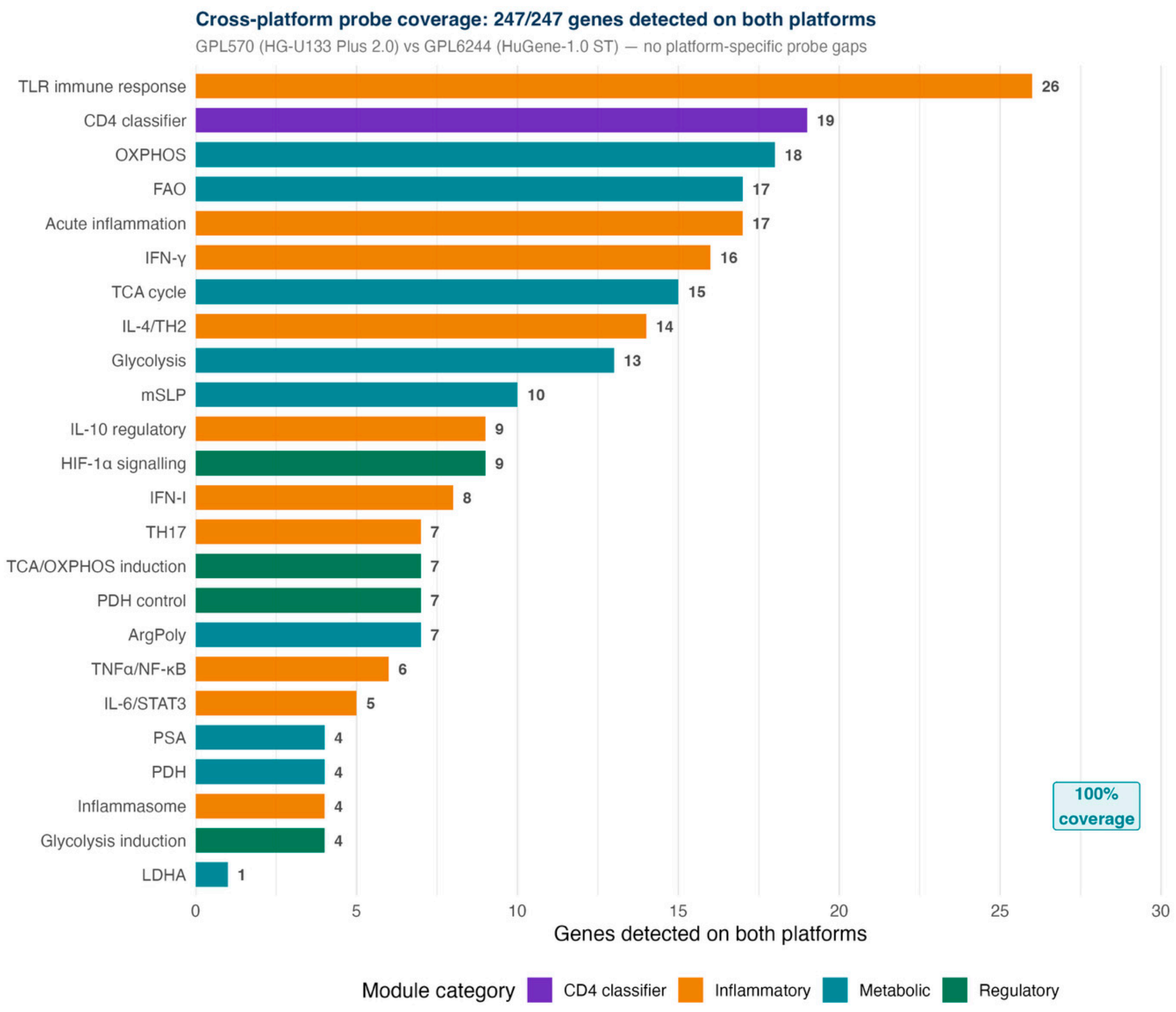

Supplementary Figure S1. Cross-platform probe coverage across immunometabolic and CD4⁺ classifier modules.

Horizontal bar chart showing the number of genes detected on both GPL570 (Affymetrix HG-U133 Plus 2.0) and GPL6244 (Affymetrix HuGene-1.0 ST) microarray platforms for each of the 24 gene modules used in this study. Bars are colour-coded by module category: $CD4^+$ classifier (purple), Inflammatory (orange), Metabolic (teal), and Regulatory (dark green). Numbers at the end of each bar indicate the total gene count per module detected on both platforms. All 247 genes across all 24 modules were successfully detected on both platforms, yielding 100% cross-platform probe coverage with no platform-specific probe gaps.
