# Supplementary material for: Immunometabolic Stratification of Autism Spectrum Disorder by CD4+ T-Cell Phenotype Reveals Subtype-Specific Energetic Deficit and Coordinated Suppression of Micronutrient Acquisition Pathways
Source: Metabolites. 2026 Jun 15;16(6):416. doi: 10.3390/metabo16060416 (PMC13304352; doi:10.3390/metabo16060416)
Supplement: Supplementary file 1 [file metabolites-16-00416-s001.zip › Supplement_S2 .pdf]

Supplementary Material S2

Immunometabolic Stratification of Autism Spectrum Disorder by CD4⁺ T Cell Phenotype Reveals Subtype-Specific Energetic Deficit and Coordinated Suppression of Micronutrient Acquisition Pathways

Albion Dervishi

Immunometabolic Module Gene Sets, Scoring Method, and Derived Metric Definitions

1. **Immunometabolic Module Gene Sets**

The 21 immunometabolic modules used in this study are defined below. Each module represents a curated gene set capturing a distinct functional pathway relevant to immune-metabolic activation. Gene membership was defined a priori based on established pathway databases (KEGG, Reactome, MSigDB) and published immunometabolic literature.

Module Name	Gene Members
**Acute Inflammation**	CXCL8, CXCL1, CXCL2, CXCL3, CCL2, CCL3, CCL4, CCL5, IL1B, IL6, TNF, ICAM1, VCAM1, PTGS2, S100A8, S100A9, NLRP3
**Arginine & Polyamine Metabolism (ArgPoly)**	GATM, ODC1, SMS, SRM, ARG1, ARG2, OAT
**Fatty Acid Oxidation (FAO)**	ACADVL, ACADL, ACAD9, ACADM, ACADS, HADHA, HADHB, HADH, ECHS1, ACAA2, ETFDH, ETFA, ETFB, CPT1A, CPT1B, CPT2, SLC25A20
**Glycolysis**	SLC2A1, SLC2A3, HK1, HK2, GPI, PFKP, ALDOA, TPI1, GAPDH, PGK1, PGAM1, ENO1, PKM
**HIF-1α Signalling**	HIF1A, ARNT, EPAS1, EGLN1, EGLN3, VEGFA, BNIP3, CA9, PDK1
**IFN-γ (IFNγ)**	HLA-DRA, HLA-DRB1, HLA-B, GBP1, GBP2, GBP4, CXCL9, CXCL10, CXCL11, IFNG, IFNGR1, IFNGR2, JAK1, JAK2, STAT1, IRF1
**Type I Interferon (IFN-I)**	IFNA1, IFNB1, IRF3, IRF7, STAT1, STAT2, IRF9, MX1, OAS1, ISG15, IFIT1
**IL-10 Regulatory Axis**	IL10, IL10RA, IL10RB, SOCS3, BCL3, NFKBIA, TNFAIP3, JAK1, TYK2, STAT3
**IL-4 / TH2**	CCL17, CCL22, IL4, IL4R, IL5, IL5RA, IL13, IL13RA1, IL13RA2, GATA3, SOCS1, IRF4, JAK1, JAK3, STAT6
**IL-6 / STAT3**	IL6, IL6R, IL6ST, CCL2, CCL20, BCL2L1, MCL1, SOCS3, JAK1, JAK2, TYK2, STAT3
**Inflammasome**	NLRP3, PYCARD, CASP1, IL1B, IL18, GSDMD
**Lactate Dehydrogenase A (LDHA)**	LDHA
**Mitochondrial Substrate-Level Phosphorylation (mSLP)**	SUCLG1, SUCLG2, SUCLA2, OGDH, DLST, DLD, GLUD1, GLUD2, GOT2, GPT2
**Oxidative Phosphorylation (OXPHOS)**	NDUFV1, NDUFS1, NDUFS2, NDUFA9, SDHA, SDHB, UQCRC1, UQCRC2, CYC1, UQCRFS1, COX4I1, COX5A, COX6C, ATP5F1A, ATP5F1B, ATP5MC1, ATP5MC2, ATP5PD
**Pyruvate Dehydrogenase Complex (PDH)**	PDHA1, PDHB, DLAT, DLD, PDHX
**Propionyl-CoA / Succinyl-CoA Anaplerosis (PSA)**	PCCB, PCCA, MCEE, MMUT

**TCA Cycle**	CS, ACO2, IDH3A, IDH3B, IDH3G, OGDH, DLST, DLD, SUCLG1, SUCLG2, SUCLA2, SDHA, SDHB, SDHC, SDHD, FH, MDH2, IDH1, IDH2, MDH1, PC, ACOD1, ACLY
**TH17**	RORC, CCR6, IL23R, STAT3, IL17A, IL17F, IL22, IL6R, IL1R1
**TLR Receptor Immune Response**	TLR1, TLR2, TLR3, TLR4, TLR5, TLR6, TLR7, TLR8, TLR9, LY96, MYD88, TIRAP, TICAM1, TICAM2, IRAK4, IRAK1, TRAF6, TRAF3, MAP3K7, TAB1, TAB2, CHUK, IKBKB, IKBKG, TBK1, MAPK8, MAPK14, NFKB1, RELA, IRF3, IRF7
**TNFα / NF-κB**	CHUK, IKBKB, MYD88, NFKB1, RELA, NFKBIA, TNFAIP3, TNF, TNFRSF1A

Table S2.1. Gene composition of the 21 immunometabolic modules used for single-sample scoring.

2. Module Scoring Method

Module activity scores were computed as the mean $\log_2$ expression of all member each above defined module gene set genes per sample, following the principle of single-sample gene set scoring established by Barbie et al. (Nature, 2009) and formalized in the GSVA framework (Hänzelmann et al., BMC Bioinformatics, 2013)[31], [32].
Formally, for module M and sample j:

$$S_j(M) = \frac{1}{|M|}\sum_{i\in M} E_{i,j}$$

where E_{ij} denotes the $\log_2$-normalized expression of gene i in sample j, and |M| is the number of genes in module M.

Unlike rank-based ssGSEA, which weights high-expressing tail genes and compares the signature ECDF to background genes, our approach uses unweighted mean expression. This simpler formulation is appropriate for curated metabolic modules in which all member genes contribute to pathway flux, and it preserves the absolute expression scale needed for ratio-based derived metrics (CECR, MECR) and cross-module summation in the τ-axis.

3. The τ-Axis: Integrated Immunometabolic Demand Index

The τ-axis is computed as the sum of mean $\log_2$ expression scores across all 21 predefined immunometabolic modules per sample:

$$\tau_j = \sum_{M\in \mathcal{P}} S_j(M)$$

For cross-dataset comparisons, τ values were z-normalized within each platform ($\tau^*_j = (\tau_j - \mu\tau) / \sigma\tau$). This formulation captures integrated immunometabolic transcriptional demand without requiring trained parameters, external weights, or reference databases. Each module contributes equally to the composite τ-axis score.

	Gene Members
**Glycolysis induction**	HIF1A, MYC, MTOR, AKT1, STAT3, NFKB1, RELA, PFKFB3
**PDH control**	PDK1, PDK2, PDK3, PDK4, PDP1, PDP2, SIRT3, PPARGC1A
**TCA / OXPHOS induction**	NRF1, TFAM, SIRT1, SIRT3, PRKAA1, PRKAA2, FOXO3, PPARGC1A, GAMT

Table S2.2. Regulatory and induction modules governing metabolic programming in CD4⁺ T cell subsets.

4. Derived Metric Definitions

All derived metrics are computed by arithmetic combination of module scores $S_j(M)$. No trained parameters, external reference databases, or weighting coefficients are required at any stage. Ratio-based metrics (CECR, MECR) use raw module scores to preserve the absolute expression scale required for meaningful division. Difference-based metrics (Gap indices, Warburg Index) operate on z-standardized scores to ensure comparability across modules with different expression ranges.

Metric	Formula	Conceptual Basis
CECR (Cytosolic Energy Compensation Ratio)	$(Glycolysis_j + LDHA_j) / (PDH_j + TCA_j + OXPHOS_j)$	Cytosolic-to-oxidative ATP production ratio; quantifies reliance on glycolytic vs. mitochondrial execution capacity
MECR (Mitochondrial Energy Compensation Ratio)	$(mSLP_j + PSA_j) / (PDH_j + TCA_j + OXPHOS_j)$	Mitochondrial bypass-to-oxidative ratio; captures substrate-level phosphorylation and anaplerotic contribution relative to full oxidative output
Warburg Index	$[z(Glycolysis_j) + z(LDHA_j)] / 2$	Standardized aerobic glycolytic bias; mean of z-scored glycolytic module and LDHA per sample
CID (Glycolytic Induction Demand)	$z(\text{Glycolysis Induction}_j)$	Transcriptional glycolytic demand; z-standardized score of the Glycolysis Induction module per sample
MIS (Metabolic Induction Score)	$z(GlyInd_j) + z(\text{TCA/OXPHOS Ind}_j) - z(\text{PDH control}_j)$	Net metabolic induction demand across glycolytic and mitochondrial axes, penalized by PDH regulatory control activity
Cytosolic Gap	$z(CECR_j) - CID_j$	Mismatch between cytosolic execution capacity and glycolytic induction demand
Warburg Gap	$\text{Warburg Index}_j - CID_j$	Mismatch between aerobic glycolytic bias and glycolytic transcriptional demand
Global Gap	$[z(CECR_j) + z(MECR_j)] / 2 - MIS_j$	Integrated energetic adequacy index; overall capacity–demand mismatch across both cytosolic and mitochondrial axes

Table S.2.3. Derived immunometabolic metrics: formulas and conceptual basis. z(·) denotes z-standardization across samples within the dataset. Subscript j denotes per-sample values.

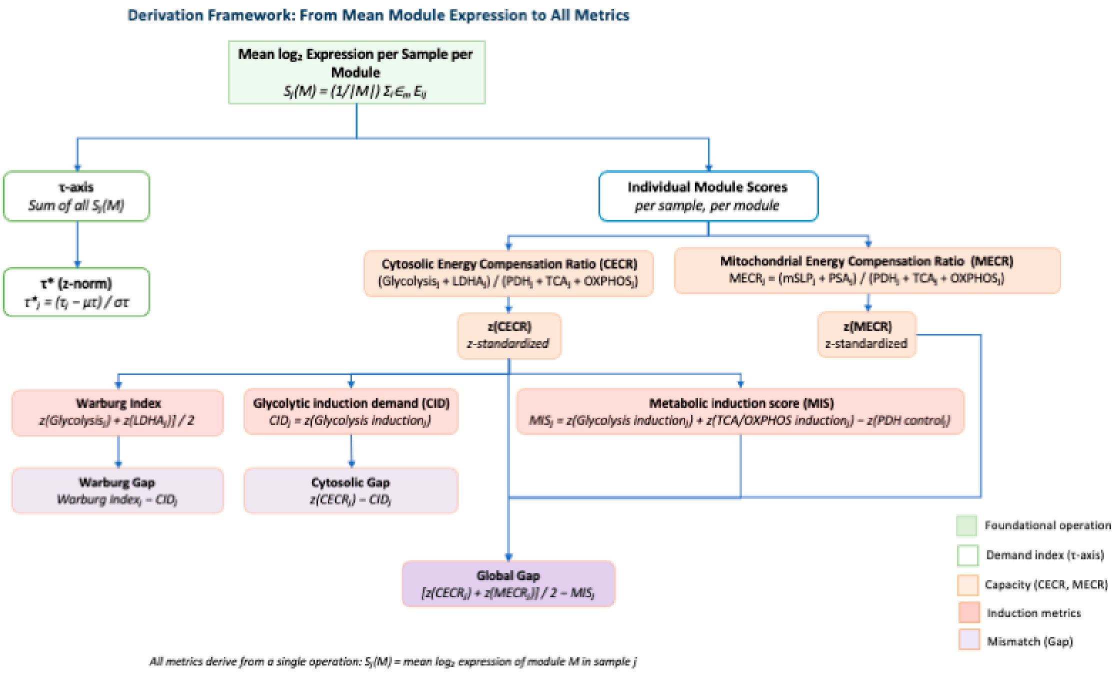

Supplementary Figure S2. Derivation hierarchy of immunometabolic metrics. All metrics derive from a single foundational operation — mean $\log_2$ expression per sample per module, $S_j(M) = (1/|M|)\ \Sigma_i \in_m E_{ij}$ — through explicit arithmetic steps requiring no trained parameters, external weights, or reference databases. CECR and MECR quantify cytosolic and mitochondrial execution capacity; CID and MIS capture transcriptional induction demand; Warburg, Cytosolic, and Global Gap metrics operationalize demand–capacity mismatch at increasing levels of integration.

[31] D. A. Barbie *et al.*, "Systematic RNA interference reveals that oncogenic KRAS-driven cancers require TBK1," *Nature*, vol. 462, no. 7269, 2009, doi: 10.1038/nature08460.

[32] S. Hänzelmann, R. Castelo, and J. Guinney, "GSVA: Gene set variation analysis for microarray and RNA-Seq data," *BMC Bioinformatics*, vol. 14, 2013, doi: 10.1186/1471-2105-14-7.
